# Assessment of Orally Administered Δ9-Tetrahydrocannabinol When Coadministered With Cannabidiol on Δ9-Tetrahydrocannabinol Pharmacokinetics and Pharmacodynamics in Healthy Adults: A Randomized Clinical Trial

**DOI:** 10.1001/jamanetworkopen.2022.54752

**Published:** 2023-02-13

**Authors:** C. Austin Zamarripa, Tory R. Spindle, Renuka Surujunarain, Elise M. Weerts, Sumit Bansal, Jashvant D. Unadkat, Mary F. Paine, Ryan Vandrey

**Affiliations:** 1 Behavioral Pharmacology Research Unit, Johns Hopkins University School of Medicine, Baltimore, Maryland; 2 Department of Pharmaceutics, School of Pharmacy, University of Washington, Seattle; 3 Center of Excellence for Natural Product Drug Interaction Research, Spokane, Washington; 4 Department of Pharmaceutical Sciences, College of Pharmacy and Pharmaceutical Sciences, Washington State University, Spokane

## Abstract

**Importance:**

Controlled clinical laboratory studies have shown that cannabidiol (CBD) can sometimes attenuate or exacerbate the effects of **Δ**9-tetrahydrocannabinol (**Δ**9-THC). No studies have evaluated differences in pharmacokinetics (PK) of **Δ**9-THC and pharmacodynamics (PD) between orally administered cannabis extracts that vary with respect to **Δ**9-THC and CBD concentrations.

**Objective:**

To compare the PK and PD of orally administered **Δ**9-THC-dominant and CBD-dominant cannabis extracts that contained the same **Δ**9-THC dose (20 mg).

**Design, Setting, and Participants:**

This randomized clinical trial was a within-participant, double-blind, crossover study conducted from January 2021 to March 2022 at the Johns Hopkins University Behavioral Pharmacology Research Unit, Baltimore, MD. Eighteen healthy adults completed 3 randomized outpatient experimental test sessions that were each separated by at least 1 week.

**Interventions:**

Brownies containing (1) no cannabis extract (ie, placebo); (2) **Δ**9-THC-dominant extract (20 mg **Δ**9-THC with no CBD); and (3) CBD-dominant extract (20 mg **Δ**9-THC + 640 mg CBD) were administered to participants 30 minutes prior to administering a cytochrome P450 (CYP) probe drug cocktail, which consisted of 100 mg caffeine, 20 mg omeprazole, 25 mg losartan, 30 mg dextromethorphan, and 2 mg midazolam.

**Main Outcomes and Measures:**

Change-from-baseline plasma concentrations for **Δ**9-THC or **Δ**9-THC metabolites and scores for subjective drug effects, cognitive and psychomotor performance, and vital signs. The area under the plasma vs concentration vs time curve (AUC) and maximum plasma concentration (C_max_) were determined.

**Results:**

The participant cohort of 18 adults included 11 males (61.1%) and 7 females (38.9%) with a mean (SD) age of 30 (7) years who had not used cannabis for at least 30 days prior to initiation of the study (mean [SD] day since last cannabis use, 86 [66] days). The CYP cocktail + placebo brownie and the CYP cocktail did not affect any PD assessments. Relative to CYP cocktail + **Δ**9-THC, CYP cocktail + **Δ**9-THC + CBD produced a higher C_max_ and area under the plasma concentration vs time curve for **Δ**9-THC, 11-OH-**Δ**9-THC, and **Δ**9-THC-COOH. The CYP cocktail + **Δ**9-THC + CBD increased self-reported anxiety, sedation, and memory difficulty, increased heart rate, and produced a more pronounced impairment of cognitive and psychomotor performance compared with both CYP cocktail + Δ9-THC and CYP cocktail + placebo.

**Conclusions and Relevance:**

In this randomized clinical trial of oral **Δ**9-THC and CBD, stronger adverse effects were elicited from a CBD-dominant cannabis extract compared with a **Δ**9-THC-dominant cannabis extract at the same **Δ**9-THC dose, which contradicts common claims that CBD attenuates the adverse effects of **Δ**9-THC. CBD inhibition of **Δ**9-THC and 11-OH-**Δ**9-THC metabolism is the likely mechanism for the differences observed. An improved understanding of cannabinoid-cannabinoid and cannabinoid-drug interactions are needed to inform clinical and regulatory decision-making regarding the therapeutic and nontherapeutic use of cannabis products.

**Trial Registration:**

clinicaltrials.gov Identifier: NCT04201197

## Key Points

**Question:**

Are there acute pharmacokinetic or pharmacodynamic differences between oral ingestion of a Δ9-tetrahydrocannabinol (Δ9-THC)-dominant cannabis extract compared with a cannabidiol (CBD)-dominant extract at the same Δ9-THC dose (20 mg) in healthy adults who use cannabis infrequently?

**Findings:**

In this randomized clinical trial including 18 adult participants, ingestion of 20 mg Δ9-THC + 640 mg CBD resulted in stronger subjective drug effects, greater impairment of cognitive and psychomotor ability, and greater increase in heart rate relative to 20 mg Δ9-THC alone and placebo. These effects appear to be mediated by CBD inhibition of Δ9-THC and 11-OH-Δ9-THC metabolism.

**Meaning:**

These findings suggest that high doses (>600 mg) of oral CBD can inhibit the metabolism of oral Δ9-THC, resulting in stronger drug effects compared with Δ9-THC in the absence of CBD.

## Teaser

This randomized clinical trial compares the pharmacokinetics and pharmacodynamics of orally administered Δ9-tetrahydrocannabinol (THC)-dominant and cannabidiol-dominant cannabis extracts that contained the same Δ9-THC dose in a healthy adult population of US adults.

## Introduction

Globally, the reform of laws regulating cannabis products and their constituent chemical components has increased access to these products and decreased the stigma associated with their use. As of this writing, 37 US states and Washington, DC, have legalized the use of cannabis for recreational and/or medicinal purposes. Collectively, more than 5 million individuals in the US have registered as medicinal cannabis users, and approximately 50 million people in the US reported using cannabis in 2020.^1^

Δ9-Tetrahydrocannabinol (Δ9-THC) and cannabidiol (CBD) are the 2 primary cannabinoids contained in cannabis products and are the most commonly purported constituents to have therapeutic benefits.^2^ Additionally, many users believe that CBD mitigates some of the adverse effects of Δ9-THC (eg, anxiogenic effects).^3^ However, results of controlled clinical laboratory research on the interactive effects of Δ9-THC and CBD were equivocal. For example, some studies have shown that CBD reduces certain acute effects associated with Δ9-THC,^4,5^ while others have shown that CBD potentiates the pharmacodynamics (PD) of Δ9-THC, resulting in stronger drug effects compared with Δ9-THC alone.^6,7^ Compounding these observations is that some studies suggest CBD alters neither the PD^8,9,10^ nor the pharmacokinetics (PK)^11,12^ of Δ9-THC.

Prior controlled Δ9-THC and CBD studies have predominantly used inhaled routes of administration, meaning much of the extant data regarding acute interactions between Δ9-THC and CBD may not translate to oral cannabis products (ie, edibles), which undergo first-pass metabolism in the intestine and liver before reaching the systemic circulation.^13^ Moreover, some prior studies have used synthetically manufactured Δ9-THC and/or CBD, limiting generality to retail oral cannabis products, which are often manufactured with whole-plant cannabis extracts. Edibles are among the most popular cannabis products and contain a large range of Δ9-THC and CBD doses that are often mislabeled.^14,15^ Additionally, evidence from the few published clinical studies suggests that Δ9-THC and CBD interact with pharmaceutical drugs, as well as with each other, via inhibition of cytochrome P450 (CYP) enzymes.^16,17,18,19^ Such inhibition can increase drug oral bioavailability and/or reduce drug clearance, which can prolong drug systemic and/or tissue concentrations and increase the risk of adverse effects.^20^

The primary goal of this study was to evaluate potential cannabinoid-drug interactions with 5 established CYP probe drugs (ie, caffeine, losartan, omeprazole, dextromethorphan, and midazolam) administered as an oral CYP cocktail, and the secondary goal (and focus of the present manuscript) was to compare the PK and PD (ie, subjective drug effects, cognitive and psychomotor performance, and vital signs) of a cannabis extract high in Δ9-THC (20 mg) and containing no CBD with an extract high in CBD containing the same dose of Δ9-THC and a therapeutically-relevant dose of CBD (640 mg).^21,22,23^ Subsequent manuscripts will describe the PK interactions between the cannabis extracts and the oral CYP cocktail probe drugs and physiologically based PK modeling and simulations resulting from this study, as those outcomes are beyond the scope of a single manuscript.

## Methods

This randomized clinical trial followed the Consolidated Standards of Reporting Trials (CONSORT) reporting guideline. This study was approved by the institutional review board at Johns Hopkins Medicine, and all participants provided written informed consent prior to study procedures.

### Participants

Study volunteers were recruited via media advertisements and word-of-mouth communication. Inclusion criteria for the study were: (1) being 18 to 50 years of age; (2) having a body mass index (BMI, calculated as weight in kilograms divided by height in meters squared) between 18 and 34; (3) past experience with cannabis; (4) no use of cannabis within 30 days prior to the first session; (5) negative urine drug test results for common drugs of abuse at screening and beginning of all sessions; (6) not pregnant or nursing; (7) report no allergies to cannabinoids or study drugs; and (8) in good health as determined via clinical chemistry blood testing (hematology and serology), medical history, and physical examination.

### Study Design and Procedures

**Figure 1.**
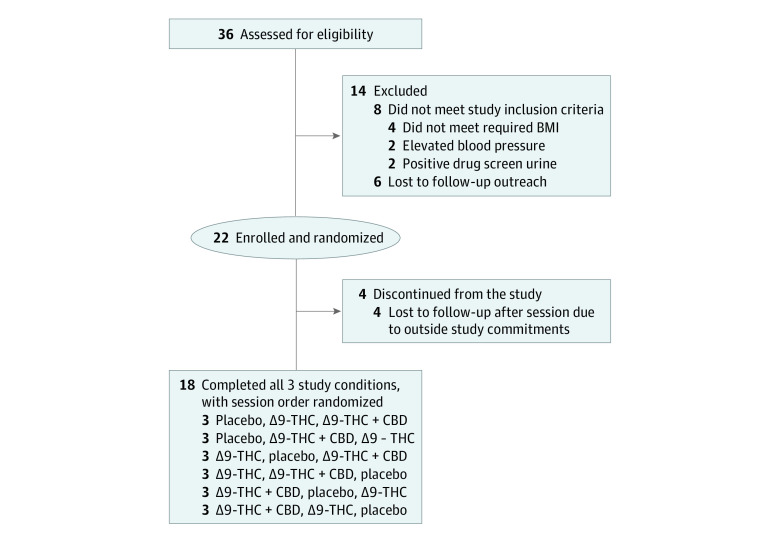
Flow Diagram of Trial Participants CBD indicates cannabidiol; THC, tetrahydrocannabinol.

This within-participant, double-blind crossover study was conducted from January 2021 to March 2022 at the Johns Hopkins Behavioral Pharmacology Research Unit. The trial protocol is available in Supplement 2. Participants who completed the study participated in three 14-hour outpatient experimental sessions during which they consumed a brownie containing vehicle (ethanol) in place of cannabis extract (placebo), a brownie containing a Δ9-THC-dominant cannabis extract with 20 mg Δ9-THC and no CBD (Δ9-THC), and a brownie containing a CBD-dominant cannabis extract with 20 mg Δ9-THC and 640 mg CBD (Δ9-THC + CBD). The doses selected were based on in vitro-in vivo modeling of interactions between Δ9-THC, CBD, and the probe drugs.^24,25^ The brownie dose condition was the primary pharmacological manipulation in the study. A randomized Latin square method was used to assign the order of brownie dose conditions to each study participant. Participants who dropped out prior to completing the study were replaced. At least 1 week separated each session to allow for drug washout (Figure 1); no data was collected during the washout periods. A pharmacist prepared and dispensed all study drugs to ensure that study staff and participants remained blinded to the experimental conditions. Thirty minutes after brownie administration, participants consumed an established CYP cocktail of 5 probe drugs: caffeine (100 mg), omeprazole (20 mg), losartan (25 mg), dextromethorphan (30 mg), and midazolam (2 mg)^26^ during all 3 experimental sessions. Serial assessments were conducted for 24 hours after CYP cocktail administration for each test session. Participants were instructed not to use any cannabis or cannabinoid products and tobacco products, caffeine, grapefruit, and other fruit juices or change any approved use of medications or dietary supplements during the study, including during the washout periods.

At the beginning of each session, participants arrived at approximately 7:30 am and consumed a standard low-fat breakfast, and an intravenous catheter was placed into a forearm vein for serial blood collection. Next, a baseline blood sample (10 mL) was collected, and baseline assessments were completed, which included the following: vital signs (heart rate [HR] and blood pressure), cognitive and psychomotor performance, and subjective drug effects. Following baseline assessments, participants consumed a brownie (approximately 1 hour after breakfast), followed by administration of the CYP cocktail approximately 30 minutes later. Participants completed PD assessments and provided blood and urine samples at timed intervals for 12 hours post-CYP cocktail administration as described in the Outcome Measures section. After 12 hours, participants were discharged home, then returned to the laboratory the next morning for a short visit for blood collections and PD assessments approximately 24 hours after CYP cocktail administration.

### Cannabis Extracts and Study Drugs

Two different whole-plant cannabis extracts were obtained from the NIDA Drug Supply Program. The Δ9-THC-dominant cannabis extract contained 70% Δ9-THC, 2.7% cannabigerol (CBG), 1.5% cannabinol (CBN), less than 1% tetrahydrocannabivarin (THCV), cannabichromene (CBC), and Δ8-THC, and no detectable CBD. The CBD-dominant cannabis extract contained 59.3% CBD, 2.1% CBC, 2.0% Δ9-THC, 1.1% CBG, and less than 1% CBN, THCV, and Δ8-THC. Both extracts were manufactured in accordance with cGMP regulations and were decarboxylated prior to use in the study. Extracts were suspended in an ethanol mixture to allow for precision with dosing, stability testing was conducted throughout the study, and sample brownies were made and tested periodically to ensure accuracy in target dosing. Placebo brownies were made with an approximately equal volume of ethanol added to the brownie mix; ethanol was allowed to dissipate from the batter prior to baking the brownies. Brownies were selected as the edible formulation based on our prior studies using this matrix to reliably deliver cannabis and be tolerated by healthy adults at the doses used.^27,28^ The CYP cocktail drugs were obtained through the Johns Hopkins Investigational Drug Services pharmacy.

### Outcome Measures

A subjective drug effect questionnaire was administered, and vitals and blood were sampled at baseline and 0.25, 0.5, 1, 2, 4, 6, 8, 12, and 24 hours after CYP cocktail administration. A battery of PD assessments was completed at baseline and 0.5, 1, 2, 3, 4, 5, 6, 8, 12, and 24 hours after CYP cocktail administration during each session.

Blood was collected into 10-mL ethylenediaminetetraacetic acid-containing vacutainer tubes and spun at 1200 g at 4 °C for 10 minutes to obtain plasma. Plasma was stored at −80 °C and shipped frozen on dry ice to the Unadkat laboratory at the University of Washington for analysis of Δ9-THC, CBD, CYP probe drugs, and corresponding metabolites. Concentrations of Δ9-THC, 11-OH-Δ9-THC, and Δ9-THC-COOH were measured using liquid chromatography-tandem mass spectrometry (LC-MS/MS) after employing a liquid-liquid extraction process.^29,30^ The limit of quantitation was 0.03, 0.1, and 0.3 ng/ml for Δ9-THC, 11-OH-Δ9-THC, and Δ9-THC-COOH, respectively. The area under the plasma concentration vs time curve (AUC) of Δ9-THC, 11-OH-Δ9-THC, and Δ9-THC-COOH for each participant within a single session was determined using the trapezoidal rule, and the maximum plasma concentration (C_max_) was documented.^31^

Subjective drug effects were assessed with the Drug Effect Questionnaire (DEQ), which has established sensitivity to the acute effects of THC and cannabis.^27,28^ DEQ items were presented on a 100 mm visual analog scale with the horizontal line anchored with 0 (not at all) and 100 (extremely). Items were assessed for 4 general categories: subjective drug effects (eg, feel drug effect), positive effects (eg, drug liking), negative/adverse effects (eg, unpleasant, anxious/nervous, sick, paranoid, sleepy, dry mouth), and perceived degree of impairment (eg, trouble with memory).

Cognitive and psychomotor performance was assessed using 3 computerized tasks that have been shown to be sensitive to acute effects of THC and cannabis^27,32,33^ and which capture various cognitive constructs (ie, psychomotor performance, working memory, divided attention) that are implicated in daily activities. Tasks included (1) the digit symbol substitution task (DSST) during which participants replicated shapes of patterns presented on their screen using a computer keyboard (primary outcome: total number correct); (2) a modified paced serial addition task (PASAT) during which participants viewed a string of single-digit numbers and attempted to select the sum of the 2 numbers most recently presented (primary outcome: total number correct); and (3) the divided attention task (DAT) during which participants track a square horizontally across the screen with a mouse cursor while simultaneously monitoring and responding to peripheral stimuli (changing numbers) that appear in the corners of the screen (primary outcome: mean distance of their cursor from the central stimulus [ie, a moving box]). During the screening evaluation, participants received training on these tasks to establish a stable baseline and minimize practice effects during sessions. The participants’ HR (ie, beats per minute; BPM) and systolic and diastolic blood pressure were measured in the seated position using an automated monitor.

### Statistical Analysis

A power analysis was conducted prior to the start of the study, which estimated that a total of 18 participants would provide 84% power to detect a 25% change in the primary outcome for this study (ie, losartan AUC), assuming 20% intra-individual variability, and a Type 1 error rate of .05. Power justification for the secondary outcomes (ie, the PD of the current report) was based on a meta-analysis comparing the statistical power of 13 drug effect assessments from 6 dose-effect studies, with 14 participants each, evaluating a range of abused drugs in our laboratory.^34^ The analysis showed that the mean effect size for secondary measures (ie, subjective drug effect ratings, behavioral or cognitive performance measures) ranged from approximately 0.87 to 1.0. Based on this estimate of effect size, the sample size of 18 was considered adequate (ie, provide the power of >0.80, or 80% chance of detecting an association, if an association exists) to assess the expected associations with PD outcomes. Demographic characteristics are presented using descriptive statistics, including mean (SD). Because there were no detectable plasma concentrations of Δ9-THC, 11-OH-Δ9-THC, or Δ9-THC-COOH after placebo brownie administration, the C_max_, the AUC of Δ9-THC and the AUC ratios of 11-OH-Δ9-THC:Δ9-THC, Δ9-THC-COOH:Δ9-THC, and Δ9-THC-COOH:11-OH-Δ9-THC were analyzed using a paired student *t* test between the CYP cocktail + Δ9-THC and the CYP cocktail + Δ9-THC + CBD conditions.

The effect of the CYP cocktail was evaluated by comparing baseline scores to postbrownie and CYP cocktail administration scores during the placebo brownie session using a separate 1-way repeated-measures analysis of variance (ANOVA) with time (0-24 hours postbrownie and CYP cocktail administration) as the primary factor. PD outcomes of the cannabis extracts were evaluated using 2-way repeated-measures ANOVAs with the within-participant factors being brownie dose condition (CYP cocktail + placebo, CYP cocktail + Δ9-THC, and CYP cocktail + Δ9-THC + CBD) and time. Planned post-hoc (Tukey) tests were conducted for peak change from baseline scores on PD assessments between each brownie dose condition (ie, CYP cocktail + placebo vs CYP cocktail + Δ9-THC, CYP cocktail + placebo vs CYP cocktail + Δ9-THC + CBD, and CYP cocktail + Δ9-THC vs CYP cocktail + Δ9-THC + CBD). For all analyses, statistical significance was defined as an α error probability of less than .05. The threshold for statistical significance for the planned contrasts was set to *P* value of less than .05, and all tests were 2-tailed. Analyses were conducted using GraphPad Prism, Version 9 (GraphPad).

## Results

Thirty-six volunteers signed informed consent. Of the 22 randomized participants, 18 (11 [61.1%] males and 7 [38.9%] females) completed the study (Figure 1). Their mean (SD) age, weight, and BMI was 30 (7) years, 78 (12) kg, and 25 (2), respectively. Self-reported race and ethnicity for individuals who completed the study included: 12 (66.7%) White and non-Hispanic individuals, 3 (16.7%) Black and non-Hispanic individuals, and 3 (16.7%) Asian and non-Hispanic individuals. At the time of study entry, a mean (SD) of 124 (80) days had passed (median [range], 93 [39-295] days) since the last self-reported cannabis use.

**Figure 2.**
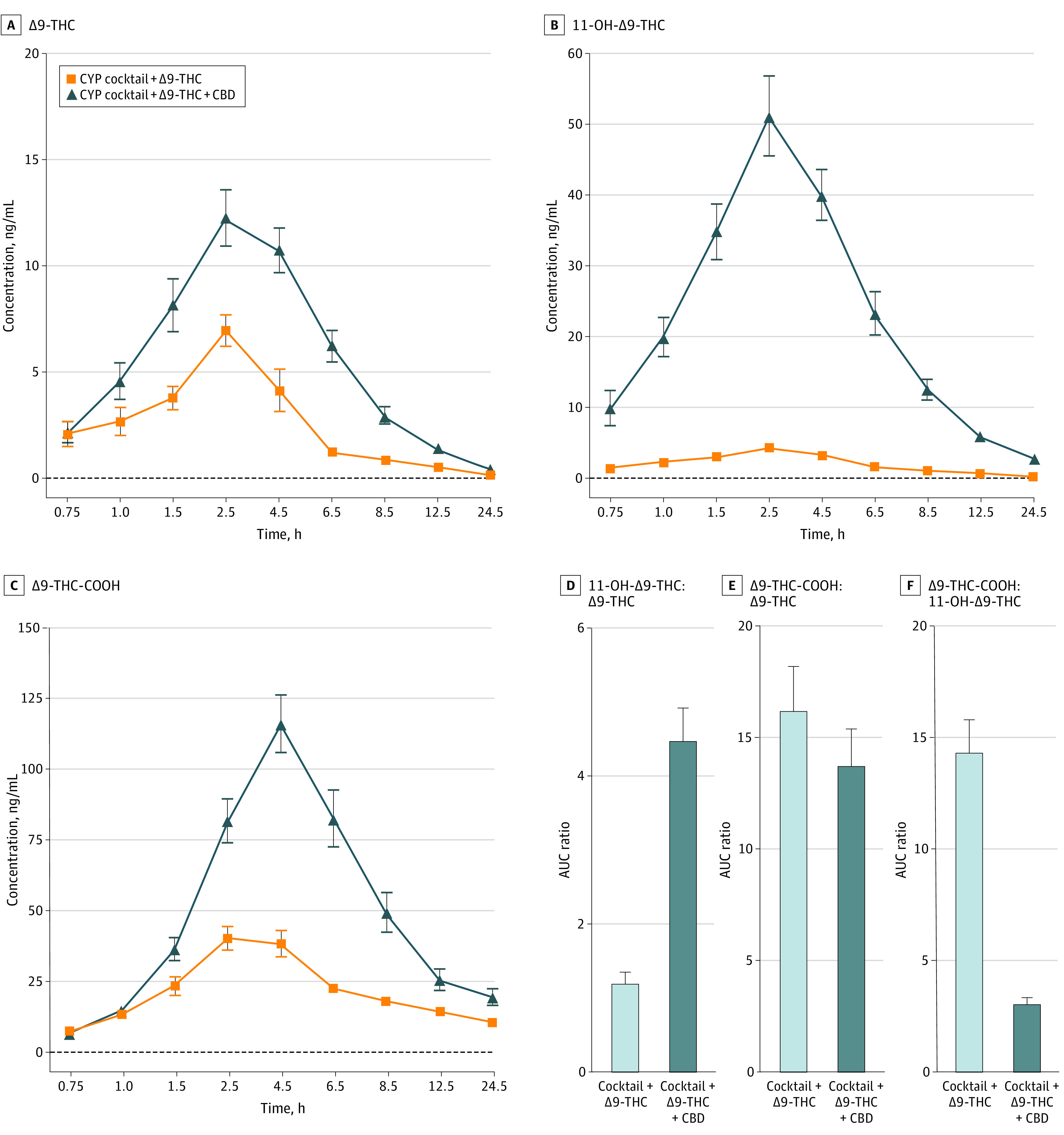
Mean Plasma Concentration vs Time Profiles for (A) Δ9-THC, (B) 11-OH-Δ9-THC, and (C) Δ9-THC-COOH Mean plasma concentration vs time profiles for (A) Δ9-THC, (B) 11-OH-Δ9-THC, and (C) Δ9-THC-COOH. Horizontal axes are not accurate time scales and represent the time points at which subjective drug effects were assessed following cannabis administration. Panels D, E, and F depict area under-the-curve ratios of 11-OH-Δ9-THC:Δ9-THC, Δ9-THC-COOH:Δ9-THC, and Δ9-THC-COOH:11-OH-Δ9-THC, respectively. Error bars indicate standard error of the means. The sample size was 18 for all outcomes. CBD indicates cannabidiol; CYP, cytochrome P450; Δ9-THC, Δ9-tetrahydrocannabinol; Δ9-THC-COOH, 11-nor-Δ9-tetrahydrocannabinol-carboxylic acid; 11-OH-Δ9-THC, 11-hydroxy-Δ9-tetrahydrocannabinol.

For plasma C_max_, Δ9-THC (Cohen *d* = 1.4 [95% CI, 0.4 to 2.4]; mean [SD], Δ9-THC + CBD, 14.8 [5.5]; Δ9-THC, 8.2 [4.0]; *P* < .001), 11-OH-Δ9-THC (Cohen *d* = 3.1 [95% CI, 1.7 to 4.4]; Δ9-THC + CBD, 53.9 [22.6]; Δ9-THC, 4.5 [1.9]; *P* < .001), and Δ9-THC-COOH (Cohen *d* = 2.1 [95% CI, 0.9 to 3.3]; Δ9-THC + CBD, 118.6 [44.8]; Δ9-THC, 45.1 [18.5]; *P* < .001) were significantly greater after CYP cocktail + Δ9-THC + CBD compared with CYP cocktail + Δ9-THC (Figure 2). Additionally, the AUC of Δ9-THC (Cohen *d* = 2.2 [95% CI, 1.0 to 3.4]; Δ9-THC + CBD, 84.9 [29.38]; Δ9-THC, 33.3 [16.9]; *P* < .001), 11-OH-Δ9-THC (Cohen *d* = 3.2 [95% CI, 1.8 to 4.6]; Δ9-THC + CBD, 349.0 [137.1]; Δ9-THC, 34.0 [16.4]; *P* < .001), and Δ9-THC-COOH (Cohen *d* = 2.8 [95% CI, 1.5 to 4.1]; Δ9-THC + CBD, 1030.0 [456.4]; Δ9-THC, 445.7 [196.2]; *P* < .001) were significantly greater after CYP cocktail + Δ9-THC + CBD compared with CYP cocktail + Δ9-THC. CYP cocktail + Δ9-THC + CBD also resulted in higher AUC ratios of 11-OH-Δ9-THC:Δ9-THC (Cohen *d* = 2.2 [95% CI, 1.1 to 3.5]; Δ9-THC + CBD, 4.5 [1.9]; Δ9-THC, 1.2 [0.7]; *P* < .001; Figure 2) and lower AUC ratios of Δ9-THC-COOH:Δ9-THC (Cohen *d* = –0.31 [95% CI, –1.2 to to 0.6]; Δ9-THC + CBD, 13.7 [7.1]; Δ9-THC, 16.2 [8.6]; *P* < .001; Figure 2) and Δ9-THC-COOH:11-OH-Δ9-THC (Cohen *d* = –2.4 [95% CI, –3.6 to –1.2]; Δ9-THC + CBD, 3.1 [1.3]; Δ9-THC, 14.4 [6.6]; *P* < .001 (Figure 2) relative to CYP cocktail + Δ9-THC (eTable 1 in Supplement 1).

**Figure 3.**
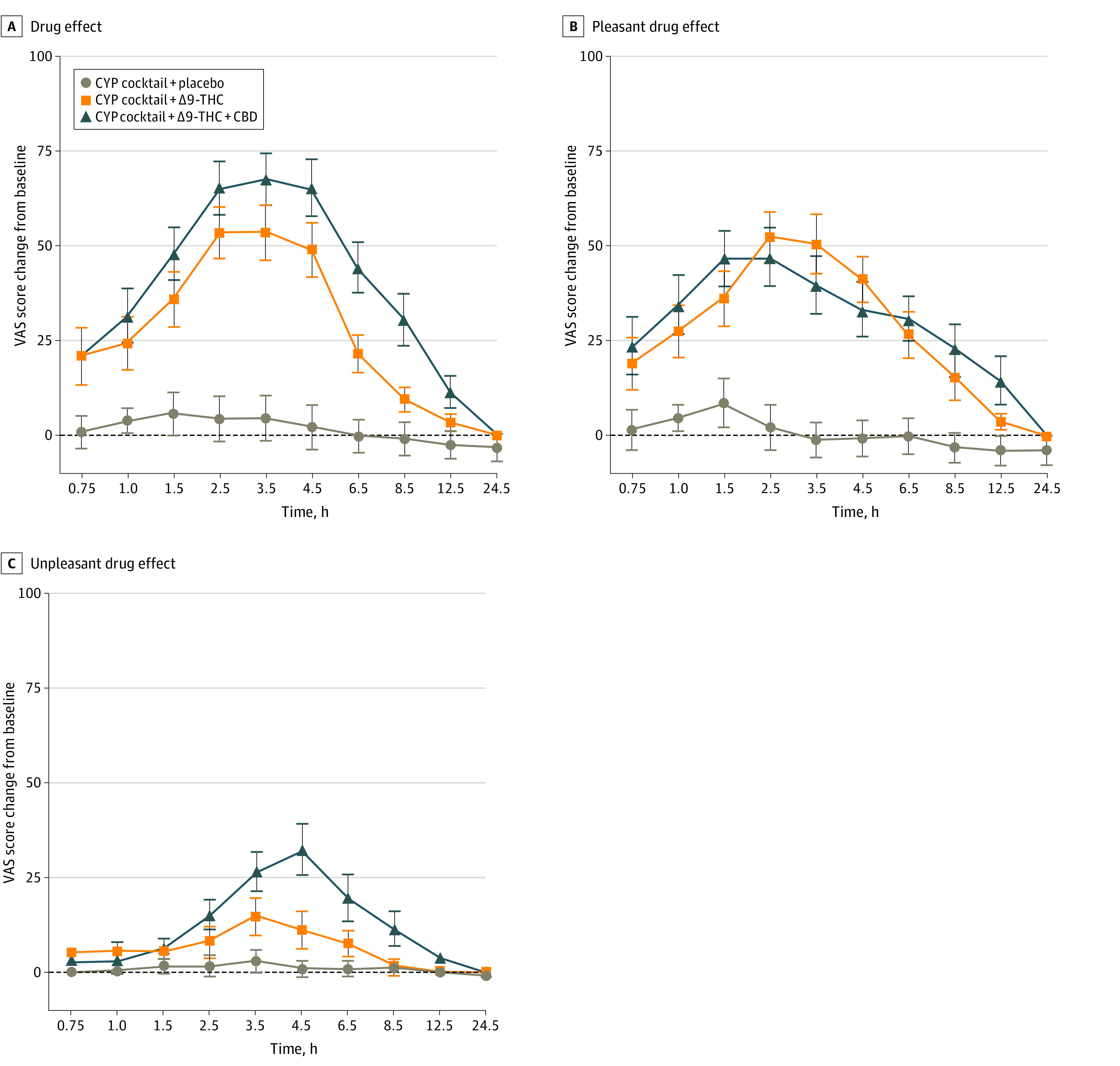
Change From Baseline Mean Ratings for the Visual Analog Scale Items (A) Drug Effect, (B) Pleasant Drug Effect, and (C) Unpleasant Drug Effect From the Drug Effect Questionnaire Displayed Over Time Scores ranged from 0 (not at all) to 100 (extremely). Error bars indicate standard error of the means. Horizontal axes are not accurate time scales and represent the time points at which subjective drug effects were assessed following cannabis administration. The sample size was 18 for all outcomes. CBD indicates cannabidiol; CYP, cytochrome P450; THC, tetrahydrocannabinol; VAS, visual analog score.

No significant effects of time were observed in the placebo brownie condition, indicating the CYP cocktail was not associated with a significant change on any of the assessed PD outcomes. The main effects of dose, time, and dose × time interactions were observed across all outcomes within the 4 subjective drug rating categories (ie, general drug effects, positive, negative/adverse, and perceived degree of impairment) (eTable 2 in Supplement 1). Planned comparisons revealed that both CYP cocktail + Δ9-THC (Cohen *d* = 1.7 [95% CI, 0.6 to 2.8]; Δ9-THC, 59.2 [31.1]; placebo, 9.4 [27.8]; *P* < .001) and CYP cocktail+Δ9-THC+CBD (Cohen *d* = 2.4 [95% CI, 1.2 to 3.6]; Δ9-THC + CBD, 72.8 [25.9]; placebo, 9.4 [27.8]; *P* < .001; *P* = .003) produced greater subjective ratings of feel drug effect than CYP cocktail + placebo (Figure 3) and that CYP cocktail + Δ9-THC + CBD produced greater ratings than CYP cocktail + Δ9-THC (Cohen *d* = 0.5 [95% CI, –0.5 to 1.4]; Δ9-THC + CBD, 72.8 [25.9]; Δ9-THC, 59.2 [31.1]; *P* < .001). Additionally, both CYP cocktail + Δ9-THC (Cohen *d* = 1.7 [95% CI, 0.6 to 2.8]; Δ9-THC, 55.9 [31.1]; placebo, 12.0 [29.4]; *P* < .001) and CYP cocktail + Δ9-THC + CBD (Cohen *d* = 2.4 [95% CI, 1.2 to 3.6]; Δ9-THC + CBD, 57.9 [29.8]; placebo, 12.0 [29.4]; *P* < .001) produced greater ratings of subjective pleasant drug effect than CYP cocktail + placebo (Figure 3) with no differences between the 2 active conditions. CYP cocktail + Δ9-THC + CBD increased subjective ratings of unpleasant (Cohen *d* = 1.7 [95% CI, 0.4 to 2.1]; Δ9-THC + CBD, 38.6 [28.0]; placebo, 4.1 [12.9]; *P* < .001) (Figure 3), anxious or nervous (Cohen *d* = 0.9 [95% CI, –0.1 to 1.8]; Δ9-THC + CBD, 23.9 [29.1]; placebo, 4.1 [14.8]; *P* = .02), sick (Cohen *d* = 0.9 [95% CI, 0.1 to 1.7]; Δ9-THC + CBD, 26.1 [22.3]; placebo, 3.4 [9.0]; *P* = .001), paranoid (Cohen *d* = 2.4 [95% CI, 1.2 to 3.6]; Δ9-THC + CBD, 23.4 [28.8]; placebo, –0.1 [8.0]; *P* = .008), red or irritated eyes (Cohen *d* = 0.6 [95% CI, –0.2 to 1.4]; Δ9-THC + CBD, 28.7 [31.6]; placebo, 3.6 [8.6]; *P* = .011), sleepy (Cohen *d* = 0.7 [95% CI, 0.1 to 1.4]; Δ9-THC + CBD, 42.6 [42.3]; placebo, –12.9 [38.3]; *P* < .001), and dry mouth (Cohen *d* = 0.9 [95% CI, 0.02 to 1.8]; Δ9-THC + CBD, 45.6 [33.5]; placebo, 1.8 [12.2]; *P* < .001) relative to CYP cocktail + placebo (Table and eFigure 1 in Supplement 1), while CYP cocktail + Δ9-THC (Cohen *d* = 0.6 [95% CI, –0.2 to 1.3]; Δ9-THC, 35.9 [36.3]; placebo, 1.8 [12.2]; *P* = .006) only increased subjective ratings of dry mouth relative to placebo. Additionally, CYP cocktail + Δ9-THC + CBD increased subjective ratings of unpleasant (Cohen *d* = 0.8 [95% CI, 0.1 to 1.5]; Δ9-THC + CBD, 38.6 [28.0]; Δ9-THC, 19.6 [23.4]; *P* = .015), sick (Cohen *d* = 0.8 [95% CI, 0.2 to 1.5]; Δ9-THC + CBD, 26.1 [22.3]; Δ9-THC, 11.6 [18.9]; *P* = .013), and red and/or irritated eyes (Cohen *d* = 1.0 [95% CI, 0.3 to 1.6]; Δ9-THC + CBD, 28.7 [31.6]; Δ9-THC, 15.9 [27.5]; *P* = .037) compared with CYP cocktail + Δ9-THC (Table). Both CYP cocktail + Δ9-THC (memory: Cohen *d* = 0.8 [95% CI, –0.06 to 1.6]; Δ9-THC, 27.4 [31.7]; placebo, 5.6 [10.0]; *P* = .01; task performance: Cohen *d* = 0.7 [95% CI, –0.01 to 1.5]; Δ9-THC, 30.3 [27.8]; placebo, 6.6 [13.4]; *P* = .005) and CYP cocktail + Δ9-THC + CBD (memory: Cohen *d* = 1.0 [95% CI, 0.01 to 1.9]; Δ9-THC + CBD, 37.8 [32.6]; placebo, 5.6 [10.0]; *P* < .001; task performance: Cohen *d* = 0.9 [95% CI, 0.01 to 1.7]; Δ9-THC + CBD, 46.7 [30.3]; placebo, 6.6 [13.4]; *P* < .001) increased subjective ratings of trouble with memory and difficulty performing routine tasks relative to CYP cocktail + placebo, and ratings of these same measures (memory: Cohen *d* = 0.3 [95% CI, –0.6 to 1.2]; Δ9-THC + CBD, 37.8 [32.6]; Δ9-THC, 27.4 [31.7]; *P* = .03; task performance: Cohen *d* = 0.6 [95% CI, –0.4 to 1.5]; Δ9-THC + CBD, 46.7 [30.3]; Δ9-THC, 30.3 [27.8]; *P* = .02) were higher for CYP cocktail + Δ9-THC + CBD compared with CYP cocktail + Δ9-THC (Table).

**Table.** Mean Peak Change From Baseline Values for Pharmacodynamic Measures^a^

Characteristics	Cocktail + 0 mg Δ9-THC + 0 mg CBD		Cocktail + 20 mg Δ9-THC + 0 mg CBD		Cocktail + 20 mg Δ9-THC + 640 mg CBD	
	Peak Change, Mean (SD)	Peak Time (Range), h	Peak Change, Mean (SD)	Peak Time (Range), h	Peak Change, Mean (SD)	Peak Time (Range), h
Subjective measures from DEQ						
Drug effect	9.4 (27.8)	1.1 (0 to 6.0)	59.2 (31.1)^b^	2.1 (0 to 4.0)	72.8 (25.9)^b^^,^^c^	2.9 (0.5 to 6.0)
Unpleasant	4.1 (12.9)	1.0 (0 to 6.0)	19.6 (23.4)^b^	1.7 (0 to 4.0)	38.6 (28.0)^b^^,^^c^	3.3 (1.0 to 6.0)
Pleasant	12.0 (29.4)	0.9 (0 to 6.0)	55.9 (31.1)^b^	2.2 (0 to 6.0)	57.9 (29.8)^b^	2.1 (0.5 to 6.0)
Drug liking	23.8 (23.2)	1.3 (0 to 4.0)	52.6 (38.3)^b^	2.2 (0 to 6.0)	60.6 (31.2)^b^	1.8 (0.5 to 4.0)
Sick	3.4 (9.0)	0.9 (0 to 6.0)	11.6 (18.9)	1.3. (0 to 6.0)	26.1 (22.3)^b^^,^^c^	3.0 (0.0 to 6.0)
Heart racing	5.3 (12.6)	0.8 (0 to 4.0)	27.7 (29.3)^b^	1.7 (0 to 6.0)	36.3 (30.7)^b^	2.1 (0.5 to 6.0)
Anxious/nervous	4.1 (14.8)	1.2 (0 to 6.0)	13.3 (20.2)	1.4 (0 to 6.0)	23.9 (29.1)^b^	2.6 (0 to 6.0)
Relaxed	– 14.3 (34.9)	2.5 (0 to 6.0)	0.3 (48.9)	2.6 (0 to 6.0)	– 11.6 (45.8)	2.2 (0 to 6.0)
Paranoid	– 0.1 (8.0)	0.4 (0 to 2.0)	12.0 (16.8)^b^	1.5 (0 to 4.0)	23.4 (28.8)^b^	2.1 (0 to 4.0)
Sleepy/tired	– 12.9 (38.3)	2.4 (0 to 6.0)	21.0 (42.9)^b^	2.6 (0 to 6.0)	42.6 (42.3)^b^	3.6 (0.5 to 6.0)
Alert	– 4.1 (30.8)	3.3 (0 to 6.0)	– 15.8 (35.4)	3.0 (0 to 6.0)	– 10.7 (44.2)	3.0 (0 to 6.0)
Irritable	– 2.1 (8.3)	0.6 (0 to 4.0)	6.7 (10.4)^b^	1.3 (0 to 6.0)	9.9 (15.6)^b^	1.9 (0 to 6.0)
Vigorous/motivated	2.3 (31.1)	2.3 (0 to 6.0)	– 2.1 (37.0)	2.1 (0 to 6.0)	– 1.7 (36.2)	1.5 (0 to 4.0)
Restless	4.2 (11.2)	0.6 (0 to 4.0)	19.4 (33.3)	2.1 (0 to 6.0)	25.2 (31.7)^b^	1.7 (0 to 6.0)
Hungry/had munchies	21.4 (32.8)	2.4 (0 to 6.0)	38.1 (34.8)	2.5 (0 to 6.0)	45.2 (40.2)	2.5 (0 to 6.0)
Cannabis craving	– 1.0 (11.5)	1.4 (0 to 6.0)	7.3 (19.2)	0.9 (0 to 3.0)	0.8 (21.2)	1.4 (0 to 6.0)
Dry mouth	1.8 (12.2)	0.9 (0 to 3.0)	35.0 (36.3)^b^	1.8 (0 to 6.0)	45.6 (33.5)^b^	3.2 (1.0 to 6.0)
Dry/red eyes	3.6 (8.6)	0.4 (0 to 1.0)	15.9 (27.5)	1.8 (0 to 4.0)	28.8 (31.6)^b^^,^^c^	2.4 (0 to 6.0)
Memory impairment	5.6 (10.0)	0.8 (0 to 4.0)	27.4 (31.7)^b^	2.2 (0 to 6.0)	37.8 (32.6)^b^^,^^c^	2.5 (0 to 4.0)
Throat irritation/coughing	2.2 (5.2)	0.5 (0 to 3.0)	10.6 (14.4)^b^	1.8 (0 to 6.0)	15.0 (19.1)^b^	2.2 (0 to 6.0)
Difficulty performing routine tasks	6.6 (13.4)	1.2 (0 to 6.0)	30.3 (27.8)^b^	1.6 (0 to 4.0)	46.7 (30.3)^b^^,^^c^	2.9 (0 to 4.0)
Cognitive measures						
DSST	2.5 (7.5)	3.6 (0.5 to 6.0)	– 2.7 (11.2)	3.3 (1.0 to 6.0)	– 9.9 (17.6)^b^	3.4 (1.0 to 6.0)
DAT	– 2.1 (9.4)	3.0 (0.5 to 6.0)	15.9 (24.7)^b^	2.2 (1.0 to 4.0)	32.7 (39.2)^b^^,^^c^	3.3 (1.0 to 6.0)
PASAT	– 1.1 (14.2)	2.8 (0.5 to 6.0)	– 6.6 (19.1)	1.9 (0.5 to 6.0)	– 22.7 (24.6)^b^^,^^c^	3.2 (1.0 to 6.0)
Physiological measures						
Heart rate, beats/min	– 4.4 (12.4)	3.3 (0.5 to 6.0)	10.1 (14.1)^b^	2.5 (0.5 to 6.0)	25.4 (20.9)^b^^,^^c^	2.3 (1.0 to 6.0)
Blood pressure, mmHg						
Diastolic	0.6 (15.3)	2.3 (0.5 to 6.0)	2.1 (17.8)	3.0 (1.0 to 6.0)	5.1 (15.5)	2.4 (0.5 to 6.0)
Systolic	3.6 (17.0)	2.6 (0.5 to 6.0)	7.4 (18.4)	2.3 (0.5 to 6.0)	– 10.8 (20.5)	2.6 (0.5 to 6.0)

Abbreviations: CBD, cannabidiol; DAT, divided attention task; DEQ, drug effect questionnaire; DSST, digit symbol substitution task; PASAT, paced serial addition task; THC, tetrahydrocannabinol.

^a^ No. = 18 for total sample.

^b^ Significant difference from placebo (*P* < .05) using a Tukey multiple comparisons test.

^c^ Significant difference between Δ-9-THC and Δ-9-THC/CBD (*P* < .05) a Tukey multiple comparisons test.

**Figure 4.**
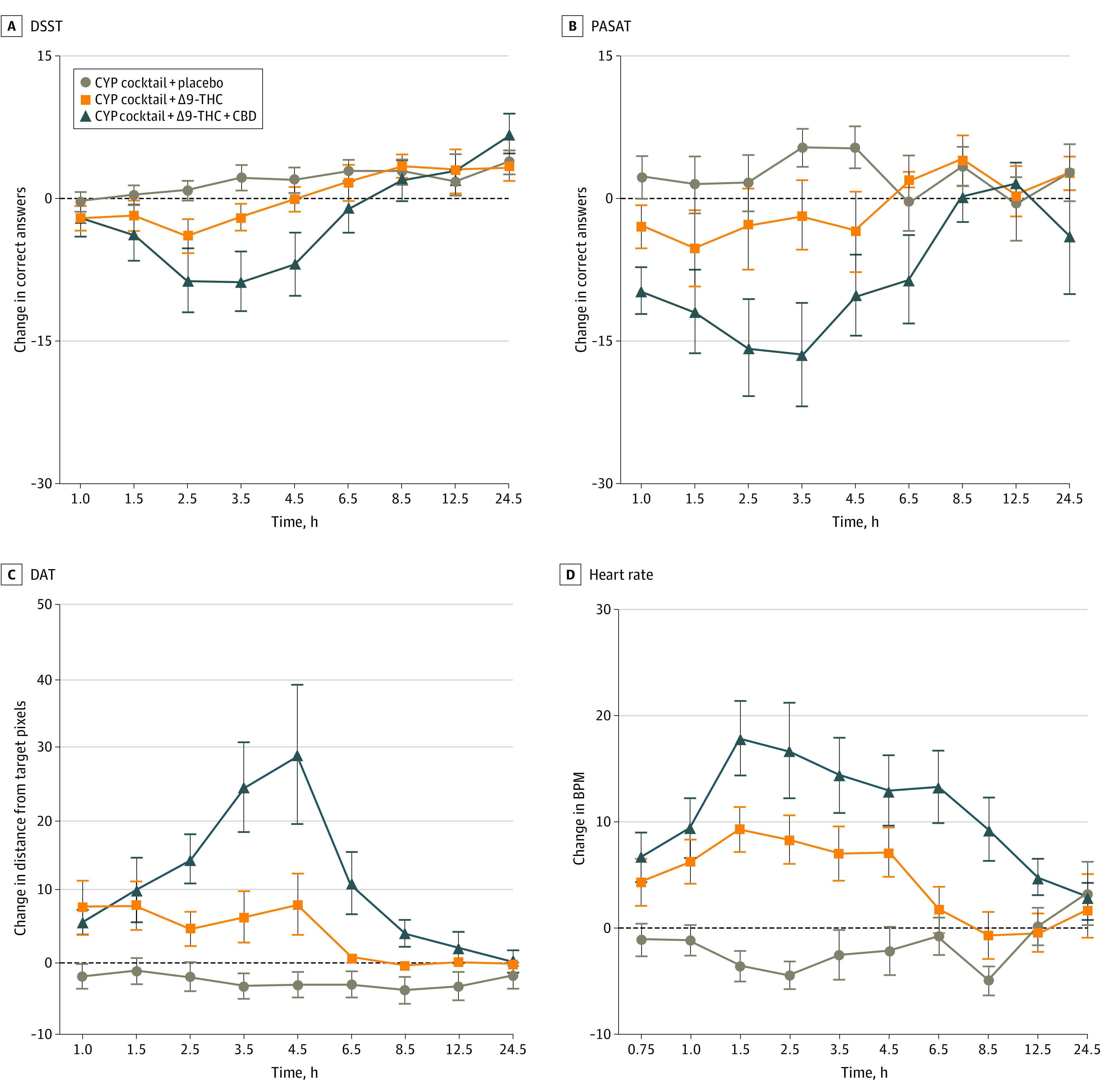
Change From Baseline Mean Total Correct on (A) the Digit Symbol Substitution Task (DSST) and (B) Paced Auditory Serial Addition Task (PASAT), the Average Distance From the Central Stimulus From the (C) Divided Attention Task (DAT), and the Beats Per Minute (BPM) for (D) Heart Rate Lower scores indicate worse performance relative to baseline in Panels A and B. Higher scores indicate worse performance relative to baseline in Panel C. Error bars indicate standard error of the means. Horizontal axes are not accurate time scales and represent the time points at which subjective drug effects were assessed following cannabis administration. The sample size was 18 for all outcomes. CBD indicates cannabidiol; CYP, cytochrome P450; Δ9-THC, Δ9-tetrahydrocannabinol.

The main effects of time and a dose × time interaction was observed for the DSST, and main effects of dose and time and a dose × time interaction were observed for the PASAT and DAT (Figure 4 and eTable 2 in Supplement 1). CYP cocktail + Δ9-THC + CBD showed significantly fewer correct trials for the DSST (Cohen *d* = –0.5 [95% CI, –1.3 to 0.2]; Δ9-THC + CBD, –9.9 [17.6]; placebo, 2.5 [7.5]; *P* = .02; Figure 4A) and PASAT (Cohen *d* = –0.7 [95% CI, –1.5 to –0.02]; Δ9-THC + CBD, –22.7 [34.6]; Δ9-THC, –1.1 [14.2]; *P* = .005; Figure 4B) compared with CYP cocktail + placebo (Table). Additionally, planned comparisons showed that CYP cocktail + Δ9-THC (Cohen *d* = 0.7 [95% CI, –0.1 to 1.5]; Δ9-THC, 15.9 [24.7]; placebo, –2.1 [9.4]; *P* = .01) and CYP cocktail + Δ9-THC + CBD (Cohen *d* = 1.0 [95% CI, –0.1 to 2.1]; Δ9-THC + CBD, 32.7 [39.2]; placebo, –2.1 [9.4]; *P* = .002) produced significantly greater distance from the central target on the DAT relative to placebo (Figure 4C). Finally, planned comparisons showed that CYP cocktail + Δ9-THC + CBD produced significantly fewer correct trials for the PASAT (Cohen *d* = –0.5 [95% CI, –1.3 to 0.2]; Δ9-THC + CBD, –22.7 [24.6]; Δ9-THC, –6.6 [19.1]; *P* = .049) and greater distance from the central target for the DAT (Cohen *d* = 0.4 [95% CI, –0.4 to 1.5]; Δ9-THC + CBD, 32.7 [39.2]; Δ9-THC, 15.9 [24.7]; *P* = .04) relative to CYP cocktail + Δ9-THC. Although DSST performance was qualitatively worse for CYP cocktail + Δ9-THC + CBD compared with CYP cocktail + Δ9-THC, the differences were not statistically significant. Main effects of dose and time and a dose × time interaction were observed for HR but not for blood pressure. HR increased more for CYP cocktail + Δ9-THC + CBD compared with both CYP cocktail + Δ9-THC (Cohen *d* = 0.7 [95% CI, –0.1 to 1.8]; Δ9-THC + CBD, 25.4 [20.9]; Δ9-THC, 10.1 [14.1]; *P* = .001) and CYP cocktail + placebo (Cohen *d* = 0.7 [95% CI, –0.1 to 1.4]; Δ9-THC + CBD, 25.4 [20.9]; placebo, –4.4 [12.4]; *P* < .001 (Figure 4). There were no effects on systolic and diastolic blood pressure (Table).

## Discussion

Given the growing popularity of cannabis edibles and oral formulations for therapeutic and nontherapeutic use, controlled research characterizing the acute PK and PD of cannabis products that vary in chemical composition other than Δ9-THC is critical. Research is particularly needed to understand the association of CBD with Δ9-THC because there is a high degree of variability in CBD content across cannabis products, and products are marketed and used based on the content and ratios of Δ9-THC:CBD. However, much of the extant data on interactions between Δ9-THC and CBD are from studies that administered the cannabis product by inhalation or Δ9-THC and CBD intravenously.^9,35,36^ The present study evaluated the association of high dose Δ9-THC when ingested alone and in combination with high dose CBD in healthy adults who were infrequent cannabis users. Overall, ingestion of Δ9-THC + CBD produced more pronounced effects, greater impairment of cognitive and psychomotor function, and increases in HR relative to both Δ9-THC and placebo. Although the doses of Δ9-THC (20 mg) and CBD (640 mg) used in the present study are somewhat high, they are within range of US Food and Drug Administration-approved therapeutic doses for prescription Δ9-THC (dronabinol) and CBD,^21,22,23^ as well as doses in products that can be purchased for medicinal use in state-regulated dispensaries.^35^

In contrast to some previous controlled studies evaluating the association of Δ9-THC or CBD alone,^9,35,36,37,38^ CBD and Δ9-THC combined produced greater increases in adverse subjective drug effect ratings and perceived impairment relative to both placebo and Δ9-THC alone.^6,7^ Further, CBD augmented Δ9-THC-induced impairment of cognitive and psychomotor ability and produced greater increases in HR relative to Δ9-THC. Discrepancies in the interactions of CBD and Δ9-THC across studies may be related to different routes of administration, CBD doses administered, or time course for evaluation. Unlike prior studies, the present study used an oral route of administration for both CBD and Δ9-THC, and in the case of the brownie high in CBD, both Δ9-THC and CBD were administered simultaneously rather than sequentially.

CBD has been shown to inhibit the cytochrome P450 enzymes (present in both the liver and intestine) which metabolize Δ9-THC and 11-OH-Δ9-THC,^24,25^ reducing the clearance of Δ9-THC and 11-OH-Δ9-THC.^16,24,25,39^ Notably, 11-OH-Δ9-THC, which is the active metabolite of Δ9-THC, has been shown to produce greater drug effects compared with Δ9-THC.^40^ The present clinical study highlights the importance of considering dose adjustments for individuals electing to use CBD-dominant vs Δ9-THC-dominant products.

### Limitations

This study has limitations. First, a single dose of Δ9-THC (20 mg) and CBD (640 mg) was administered, and a CBD only condition was lacking. Future studies comparing multiple doses and ratios of Δ9-THC and CBD are needed to determine the generality of the observed associations, as well as the dose threshold for clinically significant alterations in PD outcomes. Second, the outcomes for Δ9-THC and Δ9-THC + CBD were assessed in the context of an oral CYP cocktail. Although no adverse effects were noted when the CYP cocktail was administered after the placebo brownie, the CYP cocktail may have contributed to the behavioral outcomes observed during the active cannabis conditions. Third, although the study included both males and females, the sample size of the present study was not powered to detect potential sex differences, which have been shown to influence acute cannabis effects.^11,41,42^

## Conclusions

In this randomized clinical trial, plasma exposure to Δ9-THC, 11-OH-Δ9-THC, and Δ9-THC-COOH following ingestion of Δ9-THC + CBD was greater than that following ingestion of Δ9-THC and placebo. Consequently, participants experienced greater increases in subjective drug effects, impairment of cognitive and psychomotor performance, and HR. With legalized medicinal and nonmedicinal use of cannabis continually expanding, cannabis users and clinical or regulatory stakeholders must understand that differential chemical compositions other than Δ9-THC can significantly alter the PK and PD of cannabis products. High concentrations of CBD can inhibit Δ9-THC metabolism, which can increase the likelihood of acute adverse effects compared with the same dose of Δ9-THC in the absence of CBD.

## Notes

Supplement 1. **eTable 1. **Mean Δ9-THC, 11-OH-Δ9-THC, and Δ9-THC-COOH Maximum Concentrations, Time to Maximum Concentration, and Total Area Under-the-Curve Following Δ9-THC and Δ9-THC+CBD Administration **eTable 2. **Statistical Analyses Results for Pharmacodynamic Outcomes Using Change-From Baseline Data **eFigure. **Change From Baseline Mean Ratings for the Visual Analog Scale Items on Adverse Outcomes

Supplement 2.
Trial Protocol

Supplement 3.
Data Sharing Statement
