# Supplementary material for: Assessment of Orally Administered Δ9-Tetrahydrocannabinol When Coadministered With Cannabidiol on Δ9-Tetrahydrocannabinol Pharmacokinetics and Pharmacodynamics in Healthy Adults: A Randomized Clinical Trial
Source: JAMA Netw Open. 2023 Feb 13;6(2):e2254752. doi: 10.1001/jamanetworkopen.2022.54752 (PMC9926328; doi:10.1001/jamanetworkopen.2022.54752)
Supplement: Supplement 1. — eTable 1. Mean Δ9-THC, 11-OH-Δ9-THC, and Δ9-THC-COOH Maximum Concentrations, Time to Maximum Concentration, and Total Area Under-the-Curve Following Δ9-THC and Δ9-THC+CBD Administration eTable 2. Statistical Analyses Results for Pharmacodynamic Outcomes Using Change-From Baseline Data eFigure. Change From Baseline Mean Ratings for the Visual Analog Scale Items on Adverse Outcomes [file jamanetwopen-e2254752-s001.pdf]

Supplemental Online Content

Zamarripa CA, Spindle TR, Surujunarain R, et al. Assessment of orally administered Δ9-tetrahydrocannabinol when coadministered with cannabidiol and Δ9-tetrahydrocannabinol pharmacokinetics and pharmacodynamics in healthy adults: a randomized clinical trial. *JAMA Netw Open*. 2023;6(2):e2254752. doi:10.1001/jamanetworkopen.2022.54752

eTable 1. Mean Δ9-THC, 11-OH-Δ9-THC, and Δ9-THC-COOH Maximum Concentrations, Time to Maximum Concentration, and Total Area Under-the-Curve Following Δ9-THC and Δ9-THC+CBD Administration
eTable 2. Statistical Analyses Results for Pharmacodynamic Outcomes Using Change-From Baseline Data
eFigure. Change From Baseline Mean Ratings for the Visual Analog Scale Items on Adverse Outcomes

This supplemental material has been provided by the authors to give readers additional information about their work.

© 2023 Zamarripa CA et al. *JAMA Network Open.*

eTable 1. Mean Δ9-THC, 11-OH-Δ9-THC, and Δ9-THC-COOH Maximum Concentrations, Time to Maximum Concentration, and Total Area Under-the-Curve Following Δ9-THC and Δ9-THC+CBD Administration

	20mg Δ9-THC + 0mg CBD			20mg Δ9-THC + 640mg CBD		
	C_{max}, Mean (SD)	T_{max}, h (Range)	AUC (SD)	C_{max}, Mean (SD)	T_{max}, h (Range)	AUC (SD)
Δ9-THC	8.2 (4.0)	2.2 (0.5-4)	33.3 (16.9)	14.8 (5.5)	2.7 (1-6)	84.9 (28.4)
11-OH-Δ9-THC	4.5 (1.9)	2.2 (1-4)	34.0 (16.4)	53.9 (22.6)	2.6 (2-4)	349.0 (137.1)
Δ9-THC-COOH	45.1 (18.5)	2.6 (1-4)	445.7 (196.2)	118.6 (44.8)	4.1 (2-6)	1030.0 (456.4)

Abbreviations: SD = Standard Deviation, h = Hours

eTable 2. Statistical Analyses Results for Pharmacodynamic Outcomes Using Change-From Baseline Data

Outcome Measure	Drug Condition (D) *F*	*p*	Time (T) *F*	*p*	D x T *F*	*p*
**Subjective Measures**						
**DEQ**						
Drug Effect	25.72	<0.0001	31.19	<0.0001	12.29	<0.0001
Unpleasant	9.67	0.0012	11.03	<0.0001	5.71	0.0050
Pleasant	18.43	<0.0001	21.06	<0.0001	5.76	<0.0001
Drug Liking	11.86	0.0002	13.79	<0.0001	3.19	0.0075
Sick	4.34	0.028	4.76	0.0024	2.51	0.040
Heart Racing	9.28	0.0016	6.22	0.0023	3.4	0.013
Anxious/Nervous	5.29	0.011	4.96	0.0022	2.71	0.037
Relaxed	0.80	0.45	2.07	0.075	1.51	0.16
Paranoid	6.42	0.0065	6.31	0.004	3.13	0.045
Sleepy/Tired	11.25	0.0004	5.45	0.0004	1.38	0.22
Alert	1.16	0.33	2.29	0.07	1.18	0.32
Irritable	3.39	0.046	2.38	0.10	2.07	0.11
Vigorous/Motivated	1.44	0.25	3.32	0.02	1.05	0.40
Restless	3.27	0.051	2.80	0.031	1.51	0.18
Hungry/Had	4.49	0.026	6.78	<0.0001	2.05	0.072
Cannabis Craving	1.48	0.24	3.16	0.051	0.71	0.57
Dry Mouth	12.11	0.0004	8.11	0.0001	4.53	0.0016
Dry/Red Eyes	5.98	0.0096	6.35	0.0022	4.32	0.0045
Memory Impairment	8.34	0.0038	8.23	0.0004	5.13	0.0010
Throat Irritation/Coughing	2.30	0.13	3.81	0.018	2.02	0.11
Difficulty Performing Routine Tasks	18.38	<0.0001	11.46	<0.0001	5.25	0.002
**Cognitive Measures**						
DSST	1.97	0.17	16.89	<0.0001	5.62	<0.0001
PASAT	8.51	0.0038	3.24	0.028	2.92	0.021
DAT	14.12	0.0001	8.00	0.0015	4.70	0.0084
**Physiological Measures**						
Heart Rate, beats/min	13.50	<0.0001	3.64	0.0065	7.76	0.0002
Diastolic Blood Pressure, mmHg	0.12	0.88	5.42	<0.0001	1.034	0.41
Systolic Blood Pressure, mmHg	1.08	0.35	2.75	0.023	1.24	0.28
Abbreviations: DEQ = drug effect questionnaire, DSST = digit symbol substitution task, DAT = divided attention task, PASAT = paced serial addition task. SD = standard deviation. N=18 for total sample.						

© 2023 Zamarripa CA et al. *JAMA Network Open.*

eFigure. Change From Baseline Mean Ratings for the Visual Analog Scale Items on Adverse Outcomes

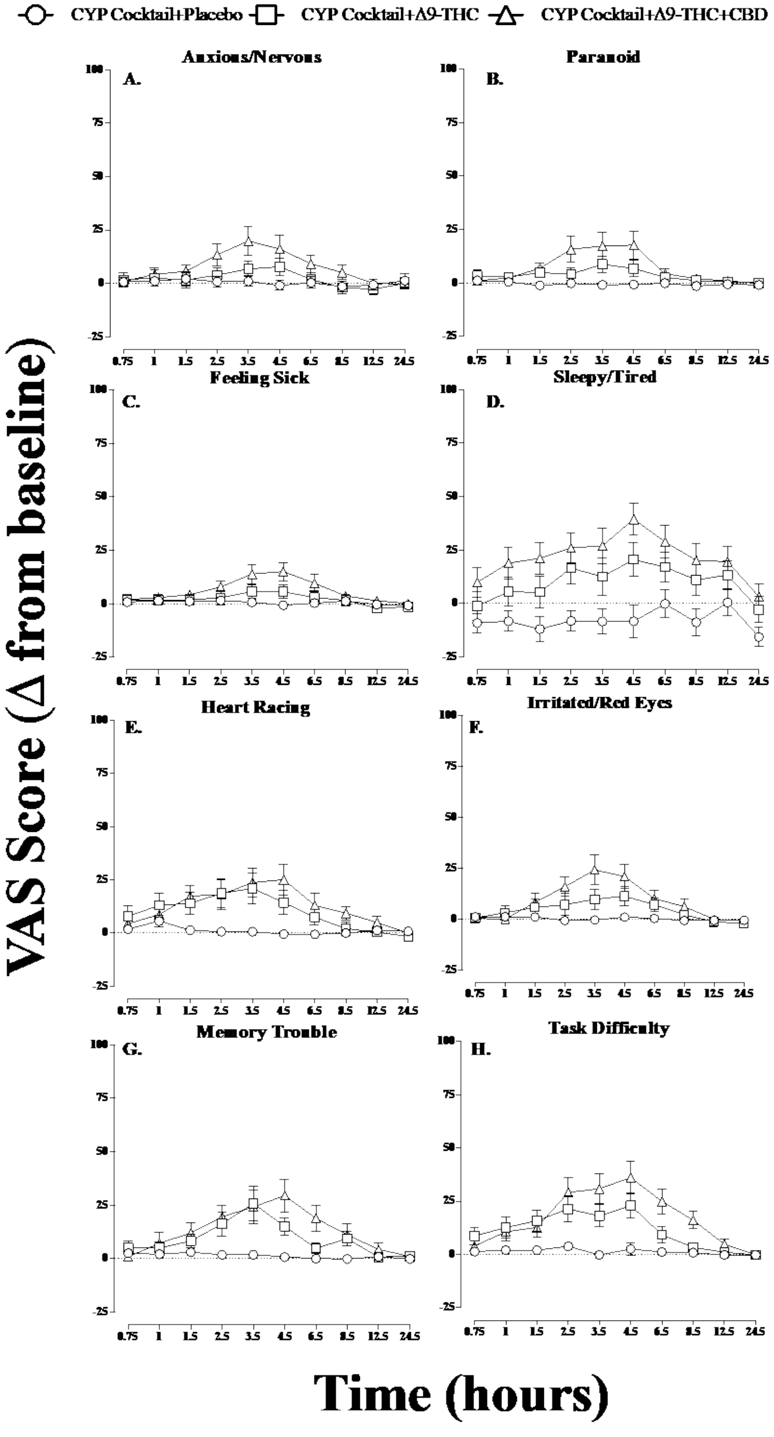

© 2023 Zamarripa CA et al. *JAMA Network Open.*
