# Supplementary material for: Assessment of Orally Administered Δ9-Tetrahydrocannabinol When Coadministered With Cannabidiol on Δ9-Tetrahydrocannabinol Pharmacokinetics and Pharmacodynamics in Healthy Adults: A Randomized Clinical Trial
Source: JAMA Netw Open. 2023 Feb 13;6(2):e2254752. doi: 10.1001/jamanetworkopen.2022.54752 (PMC9926328; doi:10.1001/jamanetworkopen.2022.54752)
Supplement: Supplement 2. — Trial Protocol [file jamanetwopen-e2254752-s002.pdf]

Date: January 22, 2021
Principal Investigator: Ryan Vandrey, PhD
Application Number: IRB00207237

JHM IRB - eForm A – Protocol

- **Use the section headings to write the JHM IRB eForm A, inserting the appropriate material in each. If a section is not applicable, leave heading in and insert N/A.**
- **When submitting JHM IRB eForm A (new or revised), enter the date submitted to the field at the top of JHM IRB eForm A.**

****

1. Abstract

a. Provide no more than a one page research abstract briefly stating the problem, the research hypothesis, and the importance of the research.

Cannabis (*aka* marijuana) and its constituents, are used to treat myriad health conditions, including pain, nausea, loss of appetite, and childhood epilepsy. Currently, 34 U.S. states and the District of Columbia have legalized the use of cannabis for medicinal and/or non-medicinal purposes, and there are now over 2 million individuals registered as medicinal cannabis users in the US. The two primary cannabinoids contained in cannabis products, and those most commonly purported to have therapeutic benefits, are delta-9-tetrahydrocannabinol (THC) and cannabidiol (CBD; Grotenhermen, 2003). In fact, most retail cannabis products are defined primarily by their THC and CBD content. Despite the widespread use and availability of cannabis products, substantive deficiencies remain regarding the potential risks for cannabis or cannabinoids to precipitate adverse interactions with conventional drugs. Evidence from the few systematic clinical studies that have been conducted suggests that THC and CBD can inhibit metabolism of other drugs, via interactions with cytochrome P450 (CYP) enzymes, a large family of enzymes involved in the metabolism of numerous drugs and foreign chemicals in the body (Benowitz et al., 1980; Geffrey et al., 2015; Yamreudeewong et al., 2009). Accordingly, evaluating the potential for drug-drug interactions between cannabis-derived products and common CYP-metabolized drugs merits further investigation. This double-blind, randomized crossover design study will evaluate whether, and to what extent, oral administration of cannabis extracts containing high doses of CBD and/or THC alter the pharmacokinetics of 5 drugs metabolized via CYP pathways including: caffeine (CYP1A2), omeprazole (CYP2C19), losartan (CYP2C9), dextromethorphan (CYP2D6), and midazolam (CYP3A). Healthy adults will complete three experimental dosing sessions, in which they will orally ingest brownies containing (1) a high THC cannabis extract with a target THC dose of 20mg, (2) a high CBD cannabis extract with a target CBD dose of 640mg + a THC dose of 20mg, or (3) placebo. In all three experimental dosing sessions, consumption of the cannabis extract infused brownie will be followed by ingestion of a drug “cocktail” comprised of commercial formulations of therapeutic or subtherapeutic doses of each drug. This collection of probe drugs, coined the Inje Cocktail, has been demonstrated to be safe, both administered alone and with various CYP450 inhibitors. At baseline and following administration of the study drugs, a battery of subjective, physiological, and cognitive performance assessments will be completed and biological specimens obtained. Each session will consist of a 12-hour outpatient drug administration visit and a 1-hour outpatient visit the subsequent day for additional biospecimen collection, cognitive testing, and subjective drug effect questionnaires. The study will conclude when 18 participants complete all 3 experimental sessions. The outcomes of this study will be useful to inform clinical decision-making regarding co-administration of cannabinoid-containing products with drugs that are either commonly prescribed by physicians or readily available over-the-counter.

2. **Objectives** (include all primary and secondary objectives)

Objective 1: Examine the influence of oral administration of cannabis products containing high doses of THC and CBD on the pharmacokinetic profile of drugs metabolized by CYP1A2, CYP2C19, CYP2C9, CYP2D6, and CYP3A enzymes.

Objective 2: Examine the acute pharmacodynamic profiles of oral THC and CBD and their relationship to pharmacokinetic outcomes.

3. **Background** (briefly describe pre-clinical and clinical data, current experience with procedures, drug or device, and any other relevant information to justify the research)

Cannabis (*aka* marijuana) and its constituents, are used widely as alternative treatments for myriad health conditions, including pain, nausea, loss of appetite, and childhood epilepsy. Researchers continue to investigate cannabis extracts/constituents as treatment for other conditions such as multiple sclerosis, seizures, AIDS, mental illnesses, and substance abuse. Currently, 34 U.S. states and the District of Columbia have legalized the use of cannabis for medicinal and/or non-medicinal purposes. Collectively, over 2 million individuals have registered as medicinal cannabis users in the U.S., and, according to the National Survey on Drug Use and Health, cannabis was used by an estimated 37.6 million users in 2016 (National Survey on Drug Use and Health, 2017).

The two primary cannabinoids contained in cannabis products, and those most commonly purported to have therapeutic benefits, include delta-9-tetrahydrocannabinol (THC) and cannabidiol (CBD; Grotenhermen, 2003). In fact, both THC and CBD are FDA approved medications (under the trade names dronabinol and epidiolex respectively) and most non-FDA approved retail cannabis products are defined primarily by their THC and CBD content. Many of these products have been manufactured to deliver very high cannabinoid doses. For example, some product lines are concentrated cannabis extracts (termed “wax”, “shatter”, “budder”, or “dabs”) that contain THC ranging from 50-90% and products containing high concentrations of CBD also abound. Food products (*aka* “edibles”) infused with cannabis have also gained widespread popularity in states where cannabis is legal (Barrus et al., 2016; Van der Pol et al., 2014). Edible cannabis products are diverse and can contain a large range of THC/CBD doses that often are mislabeled (Bonn-Miller et al., 2017; Vandrey et al., 2015). Clinical research indicates dose-dependent effects of oral cannabis, but a high degree of inter-individual variability in response to orally ingested THC, especially between tolerant and non-tolerant users and between male and female participants (Fogel et al., 2017; Schlienz et al., 2017; Vandrey et al., 2017).

Despite the widespread use and availability of cannabis products, substantive deficiencies remain regarding drug-drug interactions between cannabis or cannabinoid use and conventional pharmaceutical drugs. Evidence from the few systematic clinical studies that have been conducted suggests that THC and CBD can inhibit metabolism of other drugs, via interactions with cytochrome P450 (CYP) enzymes. In one study, CBD, when administered orally to human subjects receiving the anti-seizure drug, clobazam, led to a 500% ± 300% (mean ± SD) increase in blood plasma concentrations of the active CYP-mediated metabolite of clobazam, norclobazam (Geffrey et al., 2015). Similarly, subjects receiving CBD with oral hexobarbital exhibited 51% increases of hexobarbital in plasma and 36% decrease in oral clearance of hexobarbital (Benowitz et al., 1980). Collectively, the CBD-mediated inhibition of norclobazam (CYP2C19, CYP3A) and hexobarbital (CYP2C9, CYP2C19) metabolism appeared to be responsible for the increase in drug plasma concentrations. Beyond CBD, the package insert for dronabinol (synthetic oral THC) states that co-administration of THC with barbiturates or antipyrine can result in decreased clearance of these drugs, possibly due to competitive inhibition of metabolism (FDA, 2018). Finally, case reports involving cannabis inhalation have described prolongation of the International Normalized Ratio (INR) of warfarin, a CYP2C9 substrate, consistent with inhibition of CYP2C9 by cannabis (Yamreudeewong et al., 2009). Importantly, increased bioavailability and/or reduced clearance of drugs can prolong their effects and increase incidences of adverse effects, particularly if the drug has a narrow therapeutic window (Mangoni & Jackson, 2004).

Given the increased accessibility to THC and/or CBD-enriched products, and their potential to alter the metabolism of commonly-used drugs, evaluating the potential for drug-drug interactions between cannabis-derived products and common CYP-metabolized drugs merits further investigation. One validated approach to characterize drug-drug interactions is to administer, under controlled conditions, the drug of interest (cannabis), or placebo, in conjunction with a "cocktail" of other drugs believed to be influenced by the focal drug's presence. Subsequently, a detailed examination of the comparative pharmacokinetics of the cocktail drugs, with and without co-administration of the drug of interest, can reveal the extent to which this drug alters the metabolism/elimination of the cocktail drugs. In this study, the drug cocktail will be comprised of several drugs metabolized via distinct CYP pathways, including: caffeine (CYP1A2), omeprazole (CYP2C19), losartan (CYP2C9), dextromethorphan (CYP2D6), and midazolam (CYP3A). These drugs will be given orally, at doses that are either therapeutic or subtherapeutic. This specific cocktail of drugs, coined the "Inje Cocktail," is effective because each drug has a distinct CYP-mediated metabolic pathway and a low adverse effect profile (Ryu et al., 2007). The Inje Cocktail has been demonstrated to be safe to administer to humans, both alone and with various CYP inhibitors, in previous clinical laboratory studies (Derungs et al., 2016; Ryu et al., 2007; Williams et al., 2016).

We believe THC and CBD have the potential to interact with the drugs of the Inje cocktail, and consequently other drugs sharing the same CYP metabolic pathway, based on several factors. First, preliminary *in vitro* testing using human liver microsomes revealed that the primary metabolite of THC, 11-OH-THC, inhibited metabolic activity of CYP2C9, CYP2D6, CYP2C19; the CYP enzymes responsible for metabolizing caffeine (CYP1A2) and midazolam (CYP3A) were not examined in these preliminary tests. Second, we modeled the likelihood of THC and CBD to precipitate CYP-mediated interactions at the doses proposed in the present study using current FDA draft guidance on drug-drug interactions (FDA-CDER, 2017). Specifically, as depicted below in Tables 1 and 2, the plasma area-under-the-curve (AUC) ratio (ratio of the AUC of the object drug in the presence of inhibitor to AUC of the object drug in the absence of inhibitor) for each CYP target was calculated using published studies (Jiang et al., 2013; Yamaori et al., 2011; 2012) or inhibition potency (IC_{50}) or inhibitory constant (K_i) calculated from pre-clinical studies conducted by colleagues on this project. Preliminary studies included biorelevant intestinal solubility determination (I_g), the maximum unbound systemic concentration (C_{max}) observed previously for CBD and THC following oral administration, and the predicted unbound hepatic inlet plasma concentration ($I_{hep,u}$). Overall, these calculations revealed that, at the THC and CBD doses proposed in the present study, both cannabinoids show a strong potential for pre-systemic (i.e., first-pass) CYP2C9-mediated interactions in the liver, as indicated by predicted AUC ratios > 2. In addition, CBD showed a strong potential for pre-systemic interactions with the four other CYP enzymes of interest for this study.

Taken together, these preliminary data and calculations suggest that administration of CBD and/or THC may influence CYP-mediated metabolism for a variety of commercially available, ubiquitous drugs. Given that cannabis-containing products are readily available to an unprecedented number of individuals and are increasingly used for therapeutic and non-therapeutic purposes, it is imperative to further elucidate the drug interaction potential between THC/CBD and drugs metabolized by these 5 CYP enzymes.

TABLE 1. *Potential presystemic drug interaction targets for* **THC** *after oral administration.*

CYP	K_i or IC_{50} (μM)		Dose (40 mg)			
	Uncorrected	Binding Corrected[a]	$I_{hep,u}$ (μM)	Predicted AUC ratio (liver)[b]	I_g (μM)	Predicted AUC ratio (intestine)[c]
CYP1A2	7.5	0.375		1.05		NA
CYP2C9	0.31	0.02		2.3		NA
CYP2C19	8.55	0.43	0.02	1.0	35	NA
CYP2D6	25.5	1.28		1.0		NA
CYP3A	>100	>5		1.0		<8

[a] $f_{u,mic}$ = 0.05; [b] AUC ratio = 1+ [$I_{hep,u}$/(K_i or IC_{50})]; [c] AUC ratio = 1+ [I_g/(K_i or IC_{50})]
Assumptions: (a) $f_{u,p}$= 0.03[24]; (b) F_a = 1; (c) k_a = 0.02 min^{-1}; (d) $C_{max,u}$ = 1.91 nM[12]; (d) I_g = biorelevant solubility; (e) $I_{hep} = f_{u,p} \times \left(C_{max} + \frac{F_a \times k_a \times Dose}{Q_{hep}/R_B} \right)$, where $f_{u,p}$ denotes fraction unbound in plasma, C_{max} denotes maximum plasma concentration, F_a denotes fraction of the dose absorbed into enterocytes, k_a denotes the first-order absorption rate constant *in vivo*, Q_{hep} denotes hepatic blood flow, and R_B denotes the blood-to-plasma concentration ratio. *NA*, not applicable.

TABLE 2. *Potential presystemic drug interaction targets for* **CBD** *after oral administration.*

CYP	K_i or IC_{50} (μM)		Dose (1280 mg)			
	Uncorrected	Binding Corrected[a]	$I_{hep,u}$ (μM)	Predicted AUC ratio (liver)[b]	I_g (μM)	Predicted AUC ratio (intestine)[c]
CYP1A2	2.7	0.32		3.4		NA
CYP2C9	2.5	0.30		7.0		NA
CYP2C19	2.9	0.35	0.76	6.2	40	NA
CYP2D6	6.2	0.74		3.5		NA
CYP3A	3.8	0.46		5.0		88

[a] $f_{u,mic}$ = 0.12; [b,c] see *TABLE 1*.
Assumptions: (a) $f_{u,p}$= 0.03[24]; (b, c, e) see *TABLE* 1; (d) $C_{max,u}$ = 1.6 nM[25]. *NA*, not applicable.

4. Study Procedures

a. Study design, including the sequence and timing of study procedures (distinguish research procedures from those that are part of routine care).

Protocol Overview. The proposed study will be conducted at the Johns Hopkins Behavioral Pharmacology Research Unit (BPRU). The purpose of the study is to examine the influence of oral cannabis products (brownies) that have been made with cannabis extracts containing high concentrations of THC or THC and CBD on the pharmacokinetic profile of a “cocktail” of CYP-metabolized drugs. The focus of this project is on the evaluation of drug-drug interactions between natural products and pharmaceuticals, thus pure cannabinoid formulations or pharmaceutical cannabinoid products (e.g. dronabinol, or Epidiolex) will not be used. All procedures will be performed in a double-blind manner using a within-subject crossover design. Participants will be healthy adults who have used cannabis previously, but not in the month prior to participation. A total of 3 outpatient drug administration sessions will be conducted for each evaluable participant.

1) Placebo brownie + drug cocktail
2) Brownie containing a high THC extract (20mg THC dose) + drug cocktail
3) Brownie containing a high THC + CBD extract (20mg THC and 640mg CBD dose) + drug cocktail

Study participants will complete a battery of pharmacokinetic and pharmacodynamic outcome assessments at baseline and for 12 hours after dosing on the day of each drug administration session and during a 1-hour outpatient visit the subsequent day. Experimental dosing sessions will be separated by at least 1 week for washout of the study drugs. Prior pharmacokinetic studies conducted in our lab show that 1 week is sufficient to washout single acute cannabis doses in health adults who infrequently use cannabis. We will recruit study volunteers until 18 participants complete the protocol. Participants who drop out of the study prior to completion of all scheduled sessions will be considered “incomplete” and will be replaced. Drug administration sessions will be completed in a randomized order.

Participants. We expect the need to recruit and consent up to 50 research volunteers in order to obtain 18 study completers. We anticipate that about 50% of those screened will not be eligible or interested in the

study, and that post-randomization, some participants may drop out of the study before completion. It is estimated that we will need to randomize 25 participants to achieve 18 completers based on drop-out rates in our recent controlled cannabis administration studies.

The target demographic for study participation are healthy adults who: have a history of intentionally using cannabis, but who have not used cannabis in the month prior to study participation, and who are not currently dependent on or seeking treatment for use of cannabis or other drugs, including alcohol.

The selection of participants who have used cannabis, but are not current frequent users allows us to recruit individuals familiar with the effects of THC without issues of high levels of THC tolerance that might be present in daily users that could impact study outcomes. Any participant that reports having had an adverse reaction to cannabis that resulted in seeking medical treatment will be excluded. In our laboratory, we have previously completed two prior studies in which participants with similar drug use histories were administered THC doses up to 50mg without any significant medical concern. Healthy adult studies of CBD outside our lab have safely administered acute doses of up to 4500mg without significant medical concern.

Participant recruitment. Participants will be recruited into the study via media advertising (e.g. newspaper, internet) and word-of-mouth communication. Advertisements will seek healthy adults who occasionally use cannabis but who are not currently trying to quit. Interested participants will receive an initial screening over the telephone, that includes interviews and self-report questionnaires that provide participant information regarding health status including physical, mental health, recreational drug use history, and experience of adverse effects following cannabis use, to determine eligibility for all criteria except those which require physical evaluation. Individuals who meet initial eligibility criteria will then be scheduled for an in-person physical evaluation.

Prior to the in-person assessment assessment, written informed consent to participate in the study will be obtained. Urine specimens will be obtained and tested for evidence of recent use of commonly abused drugs. Participants must provide a government-issued photo ID confirming they are 18-50 years old, and report no allergies to cannabis or any drugs included in the Inje Cocktail. Study participants will also undergo a physical exam including clinical chemistry, hematology, serology, and serum pregnancy test (females only). Those who appear eligible for participation will receive training on the study assessment measures (e.g. exposure to subjective questionnaires and cognitive performance tasks). Participants who successfully complete training will be invited to participate in the study.

Experimental Session Procedures. For all study sessions, participants will be scheduled to arrive at approximately 7:00am on the day of drug exposure. Until such time as Covid-19-related restrictions on day-to-day operations are no longer required, social distancing will be maintained to the extent possible and all staff and study participants will be required to wear PPE throughout all face-to-face interactions. This will include mandatory use of face masks and, when closer than 6ft, use of face shields and disposable gloves. Participants will be provided PPE by study staff as necessary.

All participants will complete a breath alcohol test on arrival. Urine drug and pregnancy testing will then be conducted for all participants to test for evidence of recent illicit drug use (e.g. cannabis, cocaine, opioids) and pregnancy. Participants with a positive BAL or urine drug screen positive for any drug except THC will be sent home and the session re-scheduled. Participants who have a 2nd positive BAL, 2nd positive urine drug screen or confirmed positive pregnancy test will be immediately discharged from the study. The Time Line Follow Back (TLFB) procedure will be conducted to record substance use since the last study visit (intake assessment or prior experimental session). Concomitant medications, including vitamins and herbal supplements taken within 14 days prior to the first experimental session and throughout study participation will be recorded. Changes in medication occurring between the screening assessment and first experimental session, or between subsequent experimental sessions will be reviewed by a study investigator and medical staff prior to starting the session to ensure the volunteer is still eligible to participate.

Baseline Assessments. Prior to drug administration, the following baseline assessments will be completed: 10mL serum blood sample, vital signs (HR, BP), subjective drug effect questionnaire, and a brief cognitive performance battery (see below for details). A buccal swab will also be obtained during the first session to collect DNA for future genotyping for polymorphisms that may influence the pharmacokinetics or pharmacodynamics of the study probe drugs and/or cannabinoids.

Experimental Drug Exposure. Once baseline measures have been obtained, the BPRU pharmacy will dispense study drugs for participant self-administration. Participants will first ingest a brownie containing a cannabis extract high in THC (20mg THC dose), a cannabis extract high in THC and CBD (20mg THC and 640mg CBD dose) or placebo. Cannabis extracts were obtained from the NIDA Drug Supply Program (see below). Participants will receive each cannabis dose (one per session) in a randomized order on three separate sessions. Cannabis brownies will be prepared using individual baking trays for each dose to ensure target dose delivery. Baking will occur using a small oven located in the BPRU pharmacy and a commercial brownie mix. The mix will be prepared according to manufacturer's instructions, with a measured dose of cannabis extract added to a portion the brownie batter mixture sufficient to make one brownie with the target CBD and/or THC doses listed above. We have prepared brownies individually in this manner for previous studies (IRB00122849 and IRB00035394) and verified that this preparation method ensures that target cannabinoid doses can be reliably achieved. Study participants and research staff will be blind to dose assignment. Participants will be provided with cannabis brownies and drinking water approximately 1 hour after finishing their standardized low-fat breakfast, and will be instructed that they need to consume the brownie within 5 minutes. Thirty minutes after ingestion of the brownie, participants will ingest the "Inje" drug cocktail medications simultaneously with 250 mL of water. Participants will be given 5 minutes to ingest the "Inje" drug cocktail. Study participants and research staff will be blinded to dose assignment for all sessions. The conclusion of the "Inje" drug cocktail administration will be considered the "0 hour" by which remaining protocol assessments will be scheduled.

Post-Drug Administration Procedures.
Following the "0-hour" time point (immediately following ingestion of drug cocktail), participants will complete a battery of assessments that includes:

1) Blood (10mL) collection at 0.25, 0.5, 1, 2, 4, 6, 8, 12, and 24 hours post-exposure.
2) Subjective drug effect ratings on computerized questionnaires and vital signs assessments at 0.25, 0.5, 1, 2, 3, 4, 6, 8, 12, and 24 hours post-exposure.
3) Cognitive performance tests at 0.5, 1, 2, 3, 4, 6, 8, 12, and 24 hours post-exposure.
4) Pooled urine collection at 0-6 hours, 6-12 hours, and 12-24 hours post-exposure.

Use of medication (including caffeine, herbal/dietary supplements) or tobacco products will not be allowed during the study sessions. Study participants who regularly use tobacco products will be provided a nicotine patch upon request. Participants will be provided water, lunch, dinner, and light snacks as needed throughout the day.

Outpatient Discharge. Participants will be discharged after completing final assessments (approximately 12 hours post-drug exposure). This time point is well beyond the time that intoxicating effects of THC are expected to have subsided based on our recent studies of oral cannabis and dronabinol. If a study participant indicates the desire to be discharged from the study early, BPRU medical staff will review the self-reported rating of "drug effect" on the most recent subjective drug effect assessment, performance on the cognitive test battery, and conduct a field sobriety assessment with the participant prior to discharge. At the end of each session, study staff will compare vital signs and cognitive performance assessments with baseline data and engage in face-to-face conversation with the participant to ensure that they are fit to leave the unit. Cognitive performance tasks obtained measure psychomotor ability, working memory, higher-order cognitive functioning and attention. If the participant is able to cognitively engage with staff, vital signs are within normative range (HR < 100bpm, SYS BP < 160mmHg, DIA BP < 90mmHg), and performance is not below 10% of baseline, the participant will be cleared to leave without further evaluation. If any of these parameters are not met, medical staff will assess the participant and a formal field sobriety test will be conducted. Note, that multiple members of the BPRU cannabis lab have received formal training on administering filed sobriety tests by a Maryland State Police Drug Recognition Expert (DRE). If the participant reports a drug effect or exhibits behavior indicative of impairment/intoxication, the

participant will be asked to remain at the BPRU until the drug effect subsides and they can pass a field sobriety test. If vital signs are out of range, then medical staff will evaluate and determine whether it is safe for the participant to be discharged or remain under observation. Participants will not be allowed to drive home; instructions will be provided at the screening session regarding the need to make alternative transportation arrangements. If a participant fails to arrange a ride, taxi transportation home will be coordinated by study staff.

24-hour follow-up assessment. The day after each experimental dosing session, study participants will return for a single follow-up evaluation that includes collection of blood/urine specimens, subjective drug effect ratings, vitals, and cognitive performance.

Study Measures. A battery of measures will be used to assess participant characteristics and drug effects during the study.

Screening. Initial study screening will be completed over the telephone. During the telephone screening assessment, staff will conduct assessments to collect background demographic data (age, gender, self-reported race and ethnicity, height, and weight) and to determine study eligibility (e.g. Medical History Interview, Drug-History Questionnaire, MINI International Neuropsychiatric Interview, Time Line Follow Back (TLFB) assessment of all substance use for the prior 90 days, self-report of adverse effects following cannabis use). If the volunteer appears eligible based on this interview, a physical examination will scheduled to be completed at the BPRU by medical staff. All major organ systems, including head, eyes, ears, nose, and throat (HEENT); cardiovascular system; lungs; abdomen (liver/spleen); extremities; skin; central nervous system (CNS); musculoskeletal system, and general appearance will be assessed. Biological specimens will be tested for routine clinical chemistry, hematology, serology, serum pregnancy test (females only), and for evidence of recent illicit drug use.

Experimental Sessions. Vital signs (heart rate, systolic blood pressure (SBP), diastolic blood pressure (DBP)) will be measured in the seated position using an automated monitor.

10 milliliters of blood will be collected at the time points specified above. Participants will have the option of having an IV catheter placed at the start of each session for blood draws, or having blood drawn via venipuncture. Blood will be collected using EDTA-containing vacutainer tubes (purple-top). Blood will be spun at 1200 *g* at 4°C for 10 minutes and the plasma transferred in triplicate (~2 mL each) to orange cap cryovials for storage at -80ºC. Specimens will be shipped frozen on dry ice for quantitative analysis of caffeine, paraxanthine, losartan, E3174 (the primary CYP2C9-mediated metabolite of losartan), omeprazole, 5'-hydroxyomeprazole, dextromethorphan, dextrorphan, midazolam, 1'-hydroxymidazolam, THC, CBD, 11-OH-THC, and COOH-THC using published UPLC-MS/MS methods. The maximum volume of blood to be collected is 310 mL per participant over the course of the entire study, which is less than the volume typically collected during a single routine blood donation (473 ml).

Urine will be collected in large pooling jugs for 24 hours after each brownie/drug administration (0-6, 6-12, and 12-24 hours). Pooled urine will be measured (volume), mixed, and aliquots transferred into conical (Falcon) polypropylene tubes (4 aliquots of 2 mL each) and stored frozen at -20oC. Specimens will be shipped frozen on dry ice for quantitative analysis of the probe drugs, cannabinoids, and metabolites.

A buccal swab will be collected at baseline during the first drug exposure session, from which DNA will be extracted. Swabs will be shipped to the University of Washington for future genotyping of polymorphisms that influence the pharmacokinetics and pharmacodynamics of the probe drugs, cannabinoids, and metabolites (*e.g*., those encoding the CYPs, transporters, and nuclear receptors).

A 22-item Drug Effect Questionnaire will be used to obtain subjective ratings of intoxication. Individual items include ratings of drug effects (i.e. drug effect, pleasant drug effect, unpleasant drug effect) and behavioral/mood states often associated with cannabis intoxication (i.e. relaxed, paranoid, hungry/have munchies). Participants will rate each item using a 100mm visual analog scale (VAS) anchored with "not at all" on one end and "extremely" on the other. This questionnaire is a standard self-report drug effect assessment and has been used in all our prior and ongoing cannabis dosing studies.

A brief battery of cognitive performance assessments will be conducted on aspects of functioning known to be sensitive to the acute effects of THC and cannabis, and which are relevant to functioning in the workplace and/or in operating a motor vehicle. All participants will be trained on the performance tasks to a stable baseline level during the screening session. Tasks include the Digit Symbol Substitution Task (DSST): Participants must hand type patterns presented to them on a computer screen for 90 seconds and outcomes include accuracy and total number of patterns completed in the allotted time; and a computerized Paced Serial Addition Task (PSAT): Participants are provided a string of single digit numbers on the computer and must add the total of the prior to integers presented and respond by selecting the answer using the computer mouse on the screen, primary outcome is a summed score of the number of correct trials during the task and the Divided Attention Task (DAT): Participants simultaneously perform two different simple tasks based on visual stimuli presented on a computer screen. Primary outcome is the accuracy with which they perform the two tasks. This battery of tasks takes approximately 12 minutes to complete. Recent studies in our laboratory have shown that these 3 tasks are sensitive to cannabis dose effects.

If procedural or data collection problems arise during a session, up to 2 experimental sessions can be repeated for each study participant. Instances of this occurring will be documented and reported to the IRB during the annual continuing review submission. Repeating 1-2 sessions for a participant will keep the total amount of blood drawn to that obtained during a routine blood donation and would not otherwise pose significant risk to the participant not already present during the study.

b. Study duration and number of study visits required of research participants.

Seven study visits will be required. One visit for screening evaluation, 3 outpatient experimental drug administration sessions (lasting approximately 12 hours each), and 3 outpatient visits on the day after each drug administration session (lasting approximately 1 hour each).

c. Blinding, including justification for blinding or not blinding the trial, if applicable.

The contents of the brownies administered in this study will be double-blinded. That is standard procedure for appropriate scientific control in studies evaluating the dose effects of psychoactive drugs.

d. Justification of why participants will not receive routine care or will have current therapy stopped.

Participants in this study will be healthy volunteers. Routine care for any medical illness that may arise during participation will not be affected.

e. Justification for inclusion of a placebo or non-treatment group.

A placebo brownie (no THC, no CBD) is required to establish the pharmacokinetics of the Inje Cocktail for each individual participant. This is required in order to determine whether THC or CBD impact metabolism of the drugs in the Inje Cocktail. Moreover, placebo is needed to interpret active drug effects on pharmacodynamic outcomes. Placebo dosing provides a control for expectancy effects on subjective reports and cognitive performance as well as non-pharmacological factors such as fatigue, hunger, and learning effects on performance tasks. Placebo dosing is standard for research studies involving evaluation of acute drug effects.

f. Definition of treatment failure or participant removal criteria.

This is not a treatment study. Participants may quit participation at any time of their own volition. The study investigators will discharge study participants for failing to attend their scheduled session, failure to follow the protocol requirements, or for other reasons not known at this time.

g. Description of what happens to participants receiving therapy when study ends or if a participant's participation in the study ends prematurely.

This is not a treatment trial; there is no direct course of therapy related to the participant population being targeted. We are recruiting healthy adults with experience using cannabis and who are not seeking treatment for substance use problems. Should any report the desire for treatment they will be referred to appropriate community service centers. Premature termination of participation may result in the need to recruit additional research volunteers, but should have no impact on the study volunteer directly.

5. Inclusion/Exclusion Criteria

Participants will meet the following eligibility criteria:

<u>Inclusion Criteria</u>

1. Report prior use of cannabis
2. Be between the ages of 18 and 50
3. Be in good general health based on a physical examination (including routine blood/urine testing), medical history, and vital signs.
4. Test negative for illicit drug use and have a negative breath alcohol reading at the screening visit and at admission for each session
5. Not be pregnant or nursing (if female). All females must have a negative serum pregnancy test at the screening visit and a negative urine pregnancy test prior to each dose administration.
6. Females must agree to use birth control during the study; females using hormonal contraceptives must provide type used, the dose, and frequency of use, and use should be maintained throughout the study.
7. Have a body mass index (BMI) in the range of 18 to 34 kg/m2
8. Blood pressure at Screening Visit does not exceed a systolic blood pressure (SBP) of 150 mmHg or a diastolic blood pressure (DBP) of 90 mmHg
9. Willingness to abstain from use of caffeine and grapefruit and other citrus fruit and juices during the study.
10. Have not donated blood in the prior 30 days

<u>Exclusion Criteria</u>

1. Non-medical use of psychoactive drugs other than cannabis, nicotine, or alcohol in past month
2. History of or current evidence of significant medical or psychiatric illness judged by the investigator to put the participant at greater risk of experiencing an adverse event due to drug exposure or completion of other study procedures.
3. Use of an OTC, systemic or topical drug(s), herbal supplement(s), or vitamin(s) within 14 days, or 5 half-lives (whichever is longer), of experimental sessions; which, in the opinion of the investigator or medical staff, will interfere with the study outcomes or the safety of the participant.
4. Use of a prescription medication (with the exception of birth control prescriptions) within 14 days (or 5 half-lives) of experimental sessions; which, in the opinion of the investigator or medical staff, will interfere with the study outcomes or the safety of the subject.
5. Use of pharmaceutical cannabinoids (e.g. dronabinol, nabilone) within the past month.
6. History of clinically significant cardiac arrhythmias or vasospastic disease (e.g., Prinzmetal's angina).
7. Clinically significant impairment of kidney, liver, or thyroid function (serum creatinine >1.2 mg/ml (kidney), liver function tests >3x the upper limit of normal (alanine amino transferase >99 U/L; aspartate amino transferase > 99 U/L), and thyroid stimulating hormone > 4.2 uIU/ml), or evidence of current anemia based on blood chemistry testing.
8. Enrolled in another clinical trial or have received any drug as part of a research study within 30 days prior to dosing.
9. History of adverse events associated with the ingestion of cannabis or any medications in the Inje cocktail judged by the investigator to present an undue risk of harm to the participant.
10. Current substance use disorder of moderate or greater severity.
11. Unable to abstain from alcohol or caffeinated beverages at least 24 prior to each study session
12. Allergy to any drug included in the CYP cocktail or ingredients in the brownie.

6. Drugs/ Substances/ Devices

a. The rationale for choosing the drug and dose or for choosing the device to be used.

Cannabis extracts have been obtained specifically for use in this study from the NIDA Drug Supply Program. Two different cannabis extracts were obtained: one with high THC concentration (approximately 69%) and no detectable CBD, and one with a high concentration of CBD (approximately 59%) and low concentration of THC (approximately 1.9%). Both cannabis extracts in their raw form are highly resinous and difficult to manipulate. To ensure precision in dose measurement and to facilitate distribution of the drug in the brownies, each extract has been dissolved in ethanol. The high THC extract has been dissolved to a concentration of 6.4% THC (about 10% extract) in solution, the high CBD extract has been dissolved to a concentration of 16.7% CBD and 0.495% THC in solution.

Brownies will be made by the BPRU pharmacy. Placebo brownies will be made with just commercial brownie mix. For the non-placebo brownies, the following procedures will be followed. The commercial brownie mix will be prepared and the baking tray will be filled half way. The cannabis extracts in ethanol solution will then be layed on top of the brownie mix and placed under a hood to allow the ethanol to evaporate. Brownie mix will then be added on top of the remaining extract and the brownie baked in an individual baking tray according to manufacturer instructions. There is no requirement to pre-heat the cannabis extracts because the THC and CBD have already been decarboxylated and contain free THC and CBD. During the baking process, any residual ethanol will evaporate. The high THC extract brownie dose condition will include a total THC dose of 20mg and no CBD. The high CBD extract brownie dose condition will include a total THC dose of 20mg and 640mg CBD. There were no high CBD extracts available through NIDA that contained no THC, which would allow us to probe CBD by itself. Thus, we have decided to match the THC dose across the two active brownie conditions in order to be able to evaluate the impact of CBD on metabolism of the probe drugs. Moreover, this approach is more ecologically valid because most commercial CBD products contain low concentrations of THC.

The selection of doses was conducted to balance the study aim, participant safety and tolerability based on previous experience in the BPRU laboratory and extant literature, and ensuring that doses are ecologically valid. In our laboratory, we have safely administered oral THC doses up to 50mg (IRB00035394) to infrequent cannabis users (as will be recruited in the present study). In this study, 25mg oral THC administration to 23 healthy adults resulted in emesis to one participant and a brief period of anxiety in another. At the 50mg THC dose, one additional participant vomited. The participants who vomited experienced immediate relief of discomfort after emesis and did not have any further bouts. Both completed additional drug administration sessions, indicating that the experience was not extremely aversive. The case of anxiety was resolved within about one hour of onset without the need for medical intervention. In both the oral cannabis study as well as a recently completed study of vaporized cannabis exposure, transient experiences of dizziness, sedation and nausea were reported by a minority of participants, the effects were dose-dependent, but did not result in the need for intervention and no participants withdrew participation as a result. In fact, half of the participants who completed the oral cannabis study volunteered for and completed the protocol extention involving smoked and vaporized cannabis administration one year later (those who elected not to return did so because of work conflicts or because they moved). In addition, edible cannabis products well over 40mg are commonplace in the cannabis retail market (www.leafly.com). Overall, adverse events beyond anxiety, sedation, or nausea (vomiting in rare instances) are unlikely given the relatively safe pharmacological profile of THC (partial agonist), which has no history of being directly associated with fatalities. In cases where a participant experiences panic and or paranoid reactions, research staff will engage the person in relaxation exercises and will suspend research procedures until the volunteer has regained comfort. These types of effects are typically of short duration and our staff is well practiced in helping manage these types of effects. In the case of an extreme adverse reaction, we will call 911 for medical assistance. There are multiple physicians working in our building and affiliated with the BPRU and the Johns Hopkins Bayview ER is located directly across the street from the BPRU, so any need for urgent medical care should not go long without treatment. There have been no serious adverse events in prior acute cannabis dosing studies conducted at the BPRU.

In prior studies, CBD has shown little to no acute intoxicating effects, particularly when ingested orally. The proposed dose of CBD (640mg) has previously been well-tolerated when administered to humans and showed no signs of toxicity (Zuardi et al., 2006; 2010; Schoedel et al., 2018). The lack of adverse effects from these ostensibly high CBD doses are likely due to the fact that CBD has extremely poor bioavailability when ingested orally (Huestis, 2007). In an abuse liability study of Epidiolex, doses of 1500mg and 4500mg CBD were administered to 35 healthy adults. Adverse events were rated as mild to moderate, and there were no clinically relevant changes or clear trends in hematology, biochemistry, urinanalysis, or ECG noted among study participants that were judged to be study-related except for the increase in aspartate aminotransferase (AST) observed in 1 participant. Given that CBD has no history of being directly associated with serious adverse events or fatalities, and has a relatively poor bioavailability when ingested orally, we do not anticipate any untoward drug effects from CBD at the proposed dose of 640mg.

In this study, the Inje Cocktail will be comprised of several drugs metabolized via distinct CYP pathways. These include: caffeine (100mg), omeprazole (20mg), losartan (25mg), dextromethorphan (30mg), and midazolam (2mg). The proposed oral doses of omeprazole, losartan, dextromethorphan, and midazolam are either commensurate with typical therapeutic doses or are at a subtherapeutic level. The 100mg dose of caffeine is equivalent to approximately one cup of coffee. This specific cocktail of drugs, coined the “Inje Cocktail,” was selected because each drug has a distinct CYP-mediated metabolic pathway, meaning they are unlikely to interact with one another, and each possesses a low adverse effect profile (Ryu et al., 2007). Further, this cocktail has been safely administered to humans, both alone and with various CYP inhibitors, in previous clinical laboratory studies (Derungs et al., 2016; Ryu et al., 2007; Williams et al., 2016) without the occurrence of serious adverse events. Importantly, the CYP enzymes responsible for metabolizing these five drugs (CYP1A2, CYP2C19, CYP2C9, CYP2D6, CYP3A) are also responsible for metabolizing hundreds of other OTC or prescription medications including over half of the top 200 prescribed drugs (Zanger et al., 2008). Thus, any observed influence of THC/CBD on metabolic activity on these probe drugs in the present study would be generalizable a litany of other drugs on the market that share a common metabolic pathway and greatly inform future therapeutic decisions involving cannabis use.

If initial testing in this study indicates difficulty with dose tolerability, or unexpected adverse events that may be due to the unique combination of study drugs then we will revise the study and/or proposed doses accordingly.

b. Justification and safety information if FDA approved drugs will be administered for non-FDA approved indications or if doses or routes of administration or participant populations are changed.

THC and CBD are FDA approved as oral formulations (THC as dronabinol or Marinol; CBD as Epidiolex).

Caffeine is Generally Recognized As Safe (GRAS) by the FDA and meprazole, losartan, dextromethorphan, and midazolam are also each FDA-approved.

As detailed above, we believe that these drugs can be safely administered together at the proposed doses and the risk for serious/severe adverse events is low. Because we are administering these drugs to healthy adults and in combinations/formulations that are not FDA approved, we have submitted this protocol to the FDA to be conducted under an IND that Dr. Vandrey will sponsor.

c. Justification and safety information if non-FDA approved drugs without an IND will be administered.

N/A

Study Statistics

a. Primary outcome variable.

The primary outcome variable will be the pharmacokinetics (area under the curve; AUC) of losartan, which was selected based on predicted AUC ratios of the probe drugs and previous clinical studies suggesting inhibition of CYP2C9 by various cannabis products.

b. Secondary outcome variables.

Secondary outcome variables will include the pharmacokinetics of the four remaining probe drugs, THC, and CBD. The pharmacodynamics (i.e., vital signs, subjective drug effects, cognitive performance) associated with THC, CBD, and placebo cannabis are also secondary outcomes.

c. Statistical plan including sample size justification and interim data analysis.

The sample size estimate for this study was based on power calculations performed using extant literature and previous work at the BPRU evaluating dose effects of acute drug administration using a within-subjects design.

The treatment/placebo ratio of log-transformed AUC will be determined for losartan and the other probe drugs and compared to predefined ranges determined by the FDA for assessing drug-drug interactions. A power calculation determined that 18 participants would provide 84% power to detect a 25% change in AUC for losartan (assuming 20% intra-individual variability for losartan AUC and a Type 1 error rate of 0.05). AUC values for study drugs will be also be compared using Student's t-tests or Wilcoxon signed-rank tests as appropriate (a p-value <.05 will be considered statistically significant).

A meta-analysis was previously conducted comparing the statistical power of 13 drug effect assessments from six dose-effect studies, with 14 participants each, evaluating a range of abused drugs in our laboratory (Felch, Di Marino, and Griffiths 1996). The analysis showed that average effect size for secondary measures (i.e., subjective drug effect ratings, behavioral/cognitive performance measures) ranged from approximately 0.87 to 1.0. Based on this estimate of effect size, the proposed sample size of 18 should be adequate to assess the expected effects. This sample selection methodology has been consistent in our long history of studies investigating dose-effects comparisons of different drugs, which have demonstrated excellent external validity and have become the FDA recommended standard for human abuse liability assessment. Subjective drug effects, vital signs and cognitive performance outcomes will be assessed using multiple regression analyses appropriate for repeated measures testing based on the final characteristics of the data set (e.g. normal distribution, skewness, kurtosis).

d. Early stopping rules.

The study will be stopped if new information is learned that indicates a serious risk to study participants.

7. Risks

a. Medical risks, listing all procedures, their major and minor risks and expected frequency.

Potential risks of cannabis exposure include dizziness, change in blood pressure, red or irritated eyes, drowsiness, easy laughing, euphoria, rapid heart rate, orthostatic hypotension, dry mouth, jitters, headache, nausea, vomiting, increased appetite, perceptual difficulties, memory lapse, hallucinations, confusion, depression, paranoid reaction, depersonalization, and rash. We are not aware of any adverse effects of CBD that are not risks of using cannabis. In other studies, using comparable doses to the present study, CBD has been well tolerated, showing no signs of overt intoxication and few, mostly mild side effects. Oral CBD is also reported to have no abuse liability. Overall, we feel that the risk of serious adverse events related to cannabis exposure in this study is minimal given that participants are healthy adults and experienced cannabis users, and the THC doses we are administering are within the range

used in prior studies in our lab with healthy adults (up to 50mg THC).

Potential risks of using caffeine include nervousness and restlessness, stomach irritation, nausea, vomiting, increased heart rate and respiration, dizziness, anxiety, and dehydration. Risks of omeprazole include headache, nausea, vomiting, diarrhea, abdominal pain, constipation, and dizziness. Side effects of losartan may include dizziness, nasal congestion, back pain, upper respiratory infection, and dry cough. Dextromethorphan risks include dizziness, drowsiness, nervousness and restlessness, nausea, vomiting, abdominal pain, respiratory depression, and tachycardia. Midazolam side effects may include nervousness and restlessness, agitation, respiratory depression, irregular heart rate, blurred vision, dry mouth, dizziness, drowsiness, and anxiety. Notably, rates of side effects for these drugs are generally low and the doses we intend to use in this study are equivalent to, or below, typical therapeutic doses. Importantly, these drugs have been safely administered together in prior clinical studies without serious adverse incident, both alone and with various CYP inhibitors (Derungs et al., 2016; Ryu et al., 2007; Williams et al., 2016). Thus, we do not feel administration of THC/CBD will increase the risk profile associated with these drugs.

Venous blood sampling may cause pain, tenderness, bruising, or bleeding at the needle puncture site. Some subjects may feel transient lightheadedness or dizziness, or lose consciousness (syncope), because of anxiety and vasovagal reaction.

A further risk is that participants may mistake the proposed studies as treatment or may delay treatment seeking in order to participate, although this is unlikely since we are targeting occasional cannabis users.

Breach of confidentiality about self-reported drug use and biological tests indicating recent drug use is also a risk.

Exposure to COVID-19 is a risk.

b. Steps taken to minimize the risks.

Participants are not a "vulnerable population" as defined by human subject protection guidelines; that is, they are not minors, pregnant women, under legal coercion or restriction, or mentally impaired. They are competent adults who provide their voluntary informed consent. Participants will be recruited via media advertisements and posters that clearly state the nature and intent of the study. The consent process will inform the participant in detail of the procedures, time involvement, compensation, risk, and treatment options other than participation in our study. Particular emphasis will be given to providing information regarding the potential risks involved with taking the study drugs. Volunteers will also be instructed that they may withdraw from participation at any time without losing any of the compensation that they have earned to that point.

It is unlikely that any adverse event should arise that requires immediate medical or psychiatric treatment. However, in case of an adverse event, participants will be under the supervision of medical/nursing staff throughout the study. The medical and nursing staff at BPRU are trained in CPR and mobile emergency crash carts are available on the same corridor where all experimental procedures will be conducted. The research facility (BPRU) is located directly across the street from the Johns Hopkins Bayview Medical Center Emergency Department, and, in case of an adverse event, staff will call 911 and participants will be taken by EMTs for immediate care. The Principal Investigator will be immediately notified of any serious adverse events that arise.

If participants develop nausea or vomiting, or any other adverse effects, after drug administration, study staff will assist the affected participant(s) appropriately and contact the study PI and BPRU medical staff. Nursing staff will be on site during all experimental test sessions and a physician is always on call.

Blood collection risk will be minimized by placing an IV catheter or performing venipuncture while participants are sitting down, and by having them remain under staff observation until it is clear that no

acute adverse effects occur as a result of the procedure. The risk of infection is negligible because standard sterile techniques will be used. IV catheterization and venipuncture pose a risk of infection or thrombophlebitis, which increases with duration of placement. This risk is minimized by use of careful sterile technique, having nursing staff check the IV/venipuncture site (with prompt attention if there are clinically significant signs or symptoms such as tenderness, swelling, or redness), and flushing the IV's with saline after each blood draw. The risk of anemia is negligible because the total amount of blood to be collected (310 mL) during the entire completed study is less than the amount (473 mL) collected within one hour during a single blood donation session. The amount of blood loss will be readily replaced without harm to study participants.

All advertisements and the informed consent process will clearly indicate that this research is designated only for those not seeking treatment, that participation is not a substitute for treatment, and that participation offers no clinical benefit. They will be clearly informed that they will be asked to consume cannabis and the other study drugs during their participation. Any participant who expresses an interest in receiving immediate treatment for cannabis or other substance use will be referred to a community treatment clinic. If this occurs during the study, their participation in the study will be terminated. As previously described, participants will be instructed that should they withdraw from the study at any point to pursue treatment they will still be compensated for their participation up until that point in the study.

Participants' names will be recorded only on the screening, informed consent, and necessary medical and payment forms. Anonymous participant identification numbers will be used on all other forms and labeling of biological fluids and test results. All information gathered will be kept in locked research staff offices or file cabinets. All medical and genetic information obtained will be handled in accordance with HIPAA regulations. Only research staff will have access to participant records. The limits of confidentiality (e.g. suspected child abuse or neglect, or harm to self or others) will be discussed in detail with the participants during the informed consent process. Because this study is funded by NIH, a Certificate of Confidentiality accompanies this project to prevent disclosure of study participation to third parties.

Until risk of exposure to COVID-19 is no longer a public health concern, we will maintain social distancing throughout the study procedures to the extent possible. All staff and study participants will be required to wear PPE appropriate to the nature of the tasks being completed and distance to others (e.g. face masks when maintaining social distancing, face masks, face shields, and disposable gloves when closer than 6ft). We will also minimize the number of staff who come in contact with any single participant to the extent possible based on duties to be performed and staff availability. Our target will be to have each participant only interact with 3 staff (medical staff member for physical evaluation, nurse for blood draws, research staff member for all other procedures).

c. Plan for reporting unanticipated problems or study deviations.

Study personnel will also follow ICH regulations (detailed in *Clinical Safety Data Management, Definitions, and Standards for Expedited Reporting)* regarding reporting of adverse events and all study deviations to the IRB and study sponsor.

d. Legal risks such as the risks that would be associated with breach of confidentiality.

This study involves questions about drug use and dangerous or illegal behavior, psychiatric history, and also will obtain genetic information. If there is a breach of confidentiality, then there may be a legal risk of release of sensitive information. To reduce the likelihood of patient records disclosure we will obtain a Certificate of Confidentiality.

e. Financial risks to the participants.

This study does not involve patients receiving treatment; therefore, the financial risks are minimal. Participants will be fairly compensated for their time and effort in complying with the study protocol.

8. Benefits

a. Description of the probable benefits for the participant and for society.

The primary benefit of the proposed research is in the knowledge gained regarding the interactive drug effects between THC/CBD and drugs metabolized by various CYP enzymes. This knowledge will advance our basic scientific understanding of THC and CBD and may guide policy and therapeutic decisions related to the use of cannabis. The study will also extend the extant literature investigating the acute dose effects of oral THC and CBD, including subjective effects, cognitive performance, and their correlation with biological cannabinoid levels. Because we anticipate relatively minor risks to these cannabis experienced, healthy study participants, we feel that the proposed research has a positive risk-benefit ratio.

9. Payment and Remuneration

a. Detail compensation for participants including possible total compensation, proposed bonus, and any proposed reductions or penalties for not completing the protocol.

All participants will be compensated $30 for completing the screening assessment, $300 for completing each of 3 outpatient drug administration sessions, $50 for completing each of the 3 follow-up outpatient sessions, a $300 completion bonus resulting in $1380 of total possible earnings for completing the entire study. Compensation of this magnitude is appropriate given the length and nature of this study. Calculations are as follows:

Screening Visit:	$30
Outpatient Drug Admin. Sessions 1-3:	$300/day ($900 total)
Outpatient Follow-up Sessions 1-3:	$50/day ($150 total)
Completion Bonus:	$300
Total Compensation:	$1380

If an experimental test session has to be repeated, study volunteers will be paid an additional $300 for the outpatient drug administration day and $50 for the outpatient follow-up the following day. There will be no additional completion bonus.

10. Costs

a. Detail costs of study procedure(s) or drug (s) or substance(s) to participants and identify who will pay for them.

The only direct costs to the participants will be their transportation to and from Bayview for each study visit. That cost has been factored into the compensation for participating.

Reference List

Barrus, D.G., *et al*. Tasty THC: Promises and Challenges of Cannabis Edibles. *Methods Rep RTI Press*. (2016).

Benowitz, N.L., Nguyen, T.L., Jones, R.T., Herning, R.I. & Bachman, J. Metabolic and psychophysiologic studies of cannabidiol-hexobarbital interaction. *Clin Pharmacol Ther*. **1**: 115-120. (1980).

Bonn-Miller, Marcel O., Mallory JE Loflin, Brian F. Thomas, Jahan P. Marcu, Travis Hyke, and Ryan Vandrey. Labeling accuracy of cannabidiol extracts sold online. *Jama.* **318**: 1708-1709. (2017).

Center for Behavioral Health Statistics and Quality. Results from the 2016 National Survey on Drug Use and Health: Detailed Tables. September, 2017.

Derungs, A., *et al*. Effects of Cytochrome P450 Inhibition and Induction on the Phenotyping Metrics of the Basel Cocktail: A Randomized Crossover Study. *Clin Pharmacokinet*. **1**: 79-91. (2016).

FDA (2018). MARINOL®(Dronabinol)Capsules. https://www.accessdata.fda.gov/drugsatfda_docs/label/2006/018651s025s026lbl.pdf. Accessed on August 14, 2018

Felch, L., Di Marino, M., & Griffiths, R. (1996). A meta-analysis of psychomotor, subject- and observer-rated effects in abuse liability studies of anxiolytics. In *Problems of Drug Dependence*, Harris, L. S., Ed. Government Printing Office: Washington, DC, 1996.

Fogel,J.S.,Kelly,T.H.,Westgate,P.M.&Lile,J.A.SexdifferencesinthesubjectiveeffectsoforalDelta(9)-THC in cannabis users. *Pharmacol Biochem Behav*. 44-51. (2017).

Food and Drug Administration (FDA) Center for Drug Evaluation and Research (CDER). Invitro metabolism and transporter mediated drug-drug interaction studies guidance for Industry (Draft guidance), U.S. Food and Drug Administration, Silver Spring, MD. (2017).

Geffrey, A.L., Pollack, S.F., Bruno, P.L. & Thiele, E.A. Drug-drug interaction between clobazam and cannabidiol in children with refractory epilepsy. *Epilepsia*. **8**: 1246-1251. (2015).

Grotenhermen, F. Pharmacokinetics and pharmacodynamics of cannabinoids. *Clin Pharmacokinet*. **4**: 327- 360. (2003).

Huestis MA. Human cannabinoid pharmacokinetics. Chemistry Biodiversity. **4**:1770-804. (2007).

Jiang, R., Yamaori, S., Okamoto, Y., Yamamoto, I. & Watanabe, K. Cannabidiol is a potent inhibitor of the catalytic activity of cytochrome P450 2C19. *Drug Metab Pharmacokinet*. **4**: 332-338. (2013).

Mangoni, A. A., & Jackson, S. H. Age-related changes in pharmacokinetics and pharmacodynamics: basic principles and practical applications. *British J Clin Pharmacol*, ***57***: 6-14. (2004).

Ryu, J. Y., I. S. Song, Y. E. Sunwoo, J. H. Shon, K. H. Liu, I. J. Cha, and J. G. Shin. "Development of the “Inje cocktail” for high-throughput evaluation of five human cytochrome P450 isoforms in vivo." *Clin Pharmacol Therapeut.* **82**: 531-540. (2007).

Schlienz, N.J., Lee, D.C., Stitzer, M.L. & Vandrey, R. The effect of high-dose dronabinol (oral THC) maintenance on cannabis self-administration. *Drug Alcohol Depend*. 254-260. (2018).

Vandrey, R., *et al*. Cannabinoid Dose and Label Accuracy in Edible Medical Cannabis Products. *JAMA*. **24**: 2491-2493. (2015).

Vandrey, R., *et al*. Pharmacokinetic Profile of Oral Cannabis in Humans: Blood and Oral Fluid Disposition and Relation to Pharmacodynamic Outcomes. *J Anal Toxicol*. **2**: 83-99. (2017).

Van der Pol, P., *et al*. Cross-sectional and prospective relation of cannabis potency, dosing and smoking behaviour with cannabis dependence: an ecological study. *Addiction*. **7**: 1101-1109. (2014).

Williams, D., Tao, X., Zhu, L., Stonier, M., Lutz, J. D., Masson, E., ... & Murthy, B. Use of a cocktail probe to assess potential drug interactions with cytochrome P450 after administration of belatacept, a costimulatory immunomodulator. *British J Clin Pharmacol.* **83**: 370-380. (2017).

Yamaori, S., Okamoto, Y., Yamamoto, I. & Watanabe, K. Cannabidiol, a major phytocannabinoid, as a potent atypical inhibitor for CYP2D6. *Drug Metab Dispos*. **11**: 2049-2056. (2011).

Yamaori, S., *et al*. Comparison in the in vitro inhibitory effects of major phytocannabinoids and polycyclic aromatic hydrocarbons contained in marijuana smoke on cytochrome P450 2C9 activity. *Drug Metab Pharmacokinet*. **3**: 294-300. (2012).

Yamreudeewong,W.,Wong,H.K.,Brausch,L.M.&Pulley,K.R. Probable interaction between warfarin and marijuana smoking. *Ann Pharmacother*. **7**: 1347-1353. (2009).

Zanger, U.M., Turpeinen, M., Klein, K. and Schwab, M., Functional pharmacogenetics/genomics of human cytochromes P450 involved in drug biotransformation. *Analytical Bioanal Chem.* ***392***: 1093-1108. (2008).

Zuardi A.W., Hallak J., Dursun S.,M., Morais S.L., Sanches R.F., Musty R.E., Crippa J.A. Cannabidiol monotherapy for treatment-resistant schizophrenia. J Psychopharmacol. **5**: 683-686. (2006).

Zuardi, A. W., Crippa, J. A. S., Dursun, S. M., Morais, S. L., Vilela, J. A. A., Sanches, R. F., & Hallak, J. E. C. Cannabidiol was ineffective for manic episode of bipolar affective disorder. *J Psychopharmacology*. ***24***: 135-137. (2010).

Felch, L. J., M. E. Di Marino, and R. R. Griffiths. 1996. "A meta-analysis of psychomotor, subject- and observer-rated effects in abuse liability studies of anxiolytics. ." In *Problems of drug dependence, 1995.*, edited by L. S. Harris, 188. Washington, D.C.: Government Printing Office.
