# Supplementary material for: Assessment of Orally Administered Δ9-Tetrahydrocannabinol When Coadministered With Cannabidiol on Δ9-Tetrahydrocannabinol Pharmacokinetics and Pharmacodynamics in Healthy Adults: A Randomized Clinical Trial
Source: JAMA Netw Open. 2023 Feb 13;6(2):e2254752. doi: 10.1001/jamanetworkopen.2022.54752 (PMC9926328; doi:10.1001/jamanetworkopen.2022.54752)
Supplement: Supplement 3. — Data Sharing Statement [file jamanetwopen-e2254752-s003.pdf]

Data Sharing Statement

Data
Data available: No

Additional Information
Explanation for why data not available: The data will be made available upon reasonable request by interested parties.
